# Supplementary material for: Cryo-EM Structures of Two Bacteriophage Portal Proteins Provide Insights for Antimicrobial Phage Engineering
Source: Viruses. 2021 Dec 16;13(12):2532. doi: 10.3390/v13122532 (PMC8703570; doi:10.3390/v13122532)
Supplement: Supplementary file 1 [file viruses-13-02532-s001.zip › D_1292118365_val-report-full_P1_phicpv4.pdf]

Preliminary Full wwPDB EM Validation Report (i)

Sep 27, 2021 – 04:55 pm BST

Deposition ID : D_1292118365

This is a Preliminary Full wwPDB EM Validation Report.

This report is produced by the wwPDB Deposition System during initial deposition but before annotation of the structure.

We welcome your comments at *validation@mail.wwpdb.org*
A user guide is available at
https://www.wwpdb.org/validation/2017/EMValidationReportHelp
with specific help available everywhere you see the (i) symbol.

PRELIMINARY VALIDATION REPORT

The following versions of software and data (see references (i)) were used in the production of this report:

EMDB validation analysis	:	0.0.0.dev97
MolProbity	:	4.02b-467
Percentile statistics	:	20191225.v01 (using entries in the PDB archive December 25th 2019)
Ideal geometry (proteins)	:	Engh & Huber (2001)
Ideal geometry (DNA, RNA)	:	Parkinson et al. (1996)
Validation Pipeline (wwPDB-VP)	:	2.23.2

1 Overall quality at a glance (i)

The following experimental techniques were used to determine the structure:
ELECTRON MICROSCOPY

The reported resolution of this entry is unknown.

Percentile scores (ranging between 0-100) for global validation metrics of the entry are shown in the following graphic. The table shows the number of entries on which the scores are based.

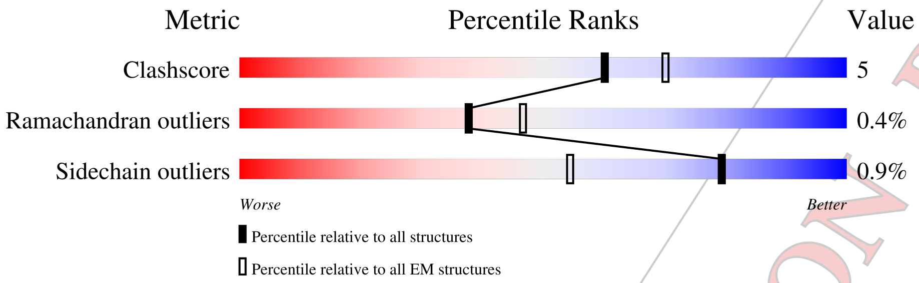

Metric	Whole archive (#Entries)	EM structures (#Entries)
Clashscore	158937	4297
Ramachandran outliers	154571	4023
Sidechain outliers	154315	3826

The table below summarises the geometric issues observed across the polymeric chains and their fit to the map. The red, orange, yellow and green segments of the bar indicate the fraction of residues that contain outliers for >=3, 2, 1 and 0 types of geometric quality criteria respectively. A grey segment represents the fraction of residues that are not modelled. The numeric value for each fraction is indicated below the corresponding segment, with a dot representing fractions <=5% The upper red bar (where present) indicates the fraction of residues that have poor fit to the EM map (all-atom inclusion < 40%). The numeric value is given above the bar.

Mol	Chain	Length	Quality of chain
1	A	256	13% / 89% 9% •
1	B	256	15% / 89% 9% •
1	C	256	12% / 89% 9% •
1	D	256	13% / 89% 9% •
1	E	256	15% / 89% 9% •
1	F	256	12% / 89% 9% •
1	G	256	13% / 89% 9% •
1	H	256	15% / 89% 9% •

Continued on next page...

PRELIMINARY VALIDATION REPORT

Continued from previous page...

Mol	Chain	Length	Quality of chain
1	I	256	12% / 89% / 9% / •
1	J	256	13% / 89% / 9% / •
1	K	256	15% / 89% / 9% / •
1	L	256	12% / 89% / 9% / •

PRELIMINARY VALIDATION REPORT

2 Entry composition (i)

There is only 1 type of molecule in this entry. The entry contains 24336 atoms, of which 0 are hydrogens and 0 are deuteriums.

In the tables below, the AltConf column contains the number of residues with at least one atom in alternate conformation and the Trace column contains the number of residues modelled with at most 2 atoms.

- Molecule 1 is a protein.

Mol	Chain	Residues	Atoms					AltConf	Trace
1	A	256	Total 2028	C 1299	N 335	O 382	S 12	0	0
1	B	256	Total 2028	C 1299	N 335	O 382	S 12	0	0
1	C	256	Total 2028	C 1299	N 335	O 382	S 12	0	0
1	D	256	Total 2028	C 1299	N 335	O 382	S 12	0	0
1	E	256	Total 2028	C 1299	N 335	O 382	S 12	0	0
1	F	256	Total 2028	C 1299	N 335	O 382	S 12	0	0
1	G	256	Total 2028	C 1299	N 335	O 382	S 12	0	0
1	H	256	Total 2028	C 1299	N 335	O 382	S 12	0	0
1	I	256	Total 2028	C 1299	N 335	O 382	S 12	0	0
1	J	256	Total 2028	C 1299	N 335	O 382	S 12	0	0
1	K	256	Total 2028	C 1299	N 335	O 382	S 12	0	0
1	L	256	Total 2028	C 1299	N 335	O 382	S 12	0	0

PRELIMINARY VALIDATION REPORT

3 Residue-property plots (i)

These plots are drawn for all protein, RNA, DNA and oligosaccharide chains in the entry. The first graphic for a chain summarises the proportions of the various outlier classes displayed in the second graphic. The second graphic shows the sequence view annotated by issues in geometry and atom inclusion in map density. Residues are color-coded according to the number of geometric quality criteria for which they contain at least one outlier: green = 0, yellow = 1, orange = 2 and red = 3 or more. A red diamond above a residue indicates a poor fit to the EM map for this residue (all-atom inclusion < 40%). Stretches of 2 or more consecutive residues without any outlier are shown as a green connector. Residues present in the sample, but not in the model, are shown in grey.

- Molecule 1:

Chain A:

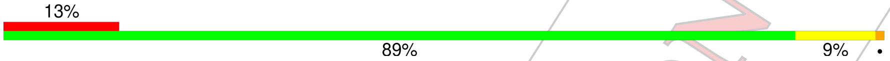

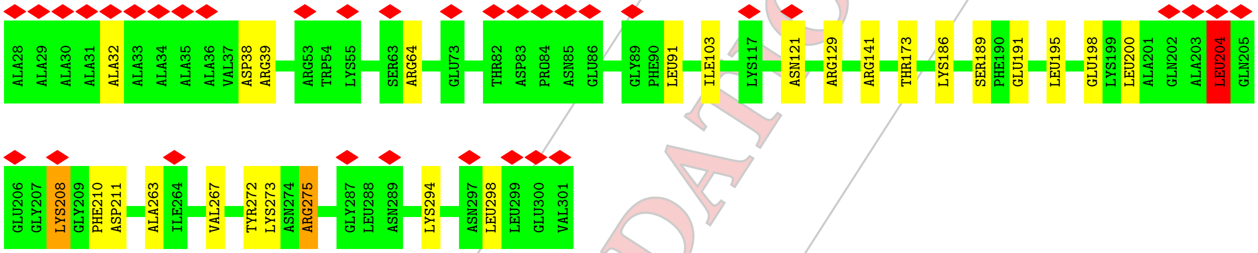

- Molecule 1:

Chain B:

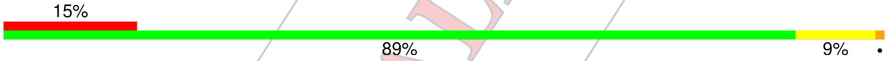

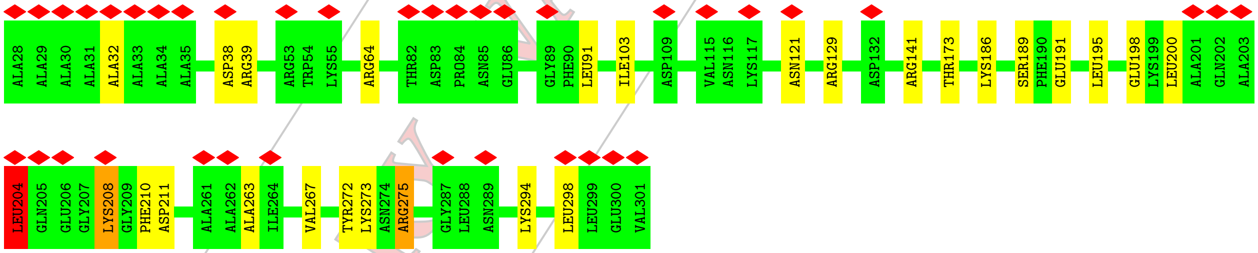

- Molecule 1:

Chain C:

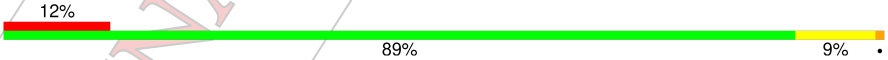

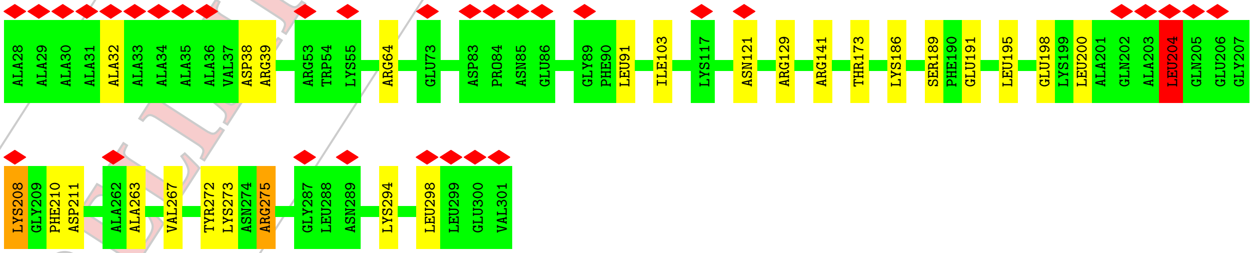

- Molecule 1:

PRELIMINARY VALIDATION REPORT

Chain D:

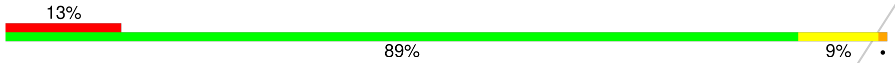

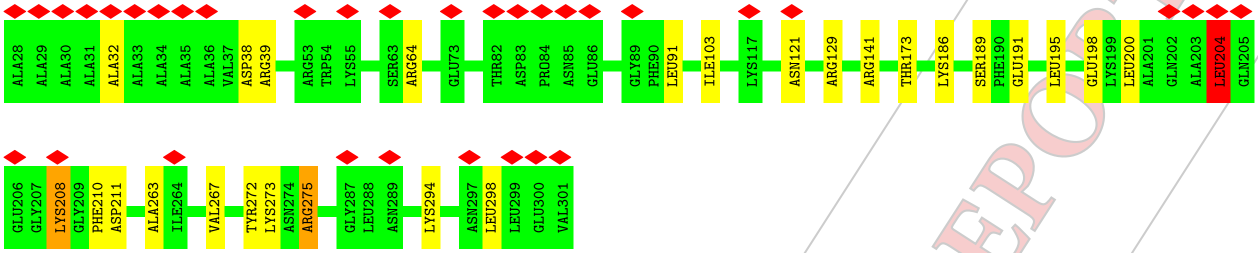

• Molecule 1:

Chain E:

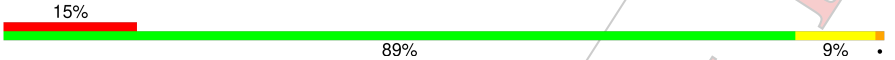

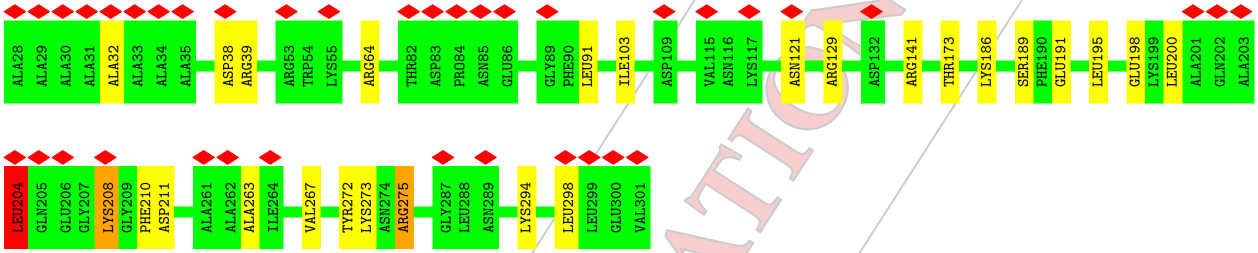

• Molecule 1:

Chain F:

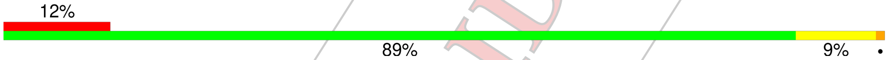

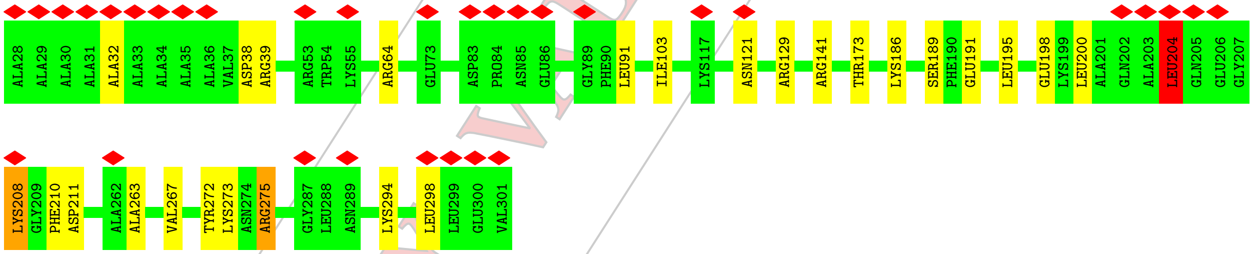

• Molecule 1:

Chain G:

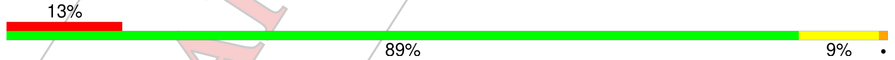

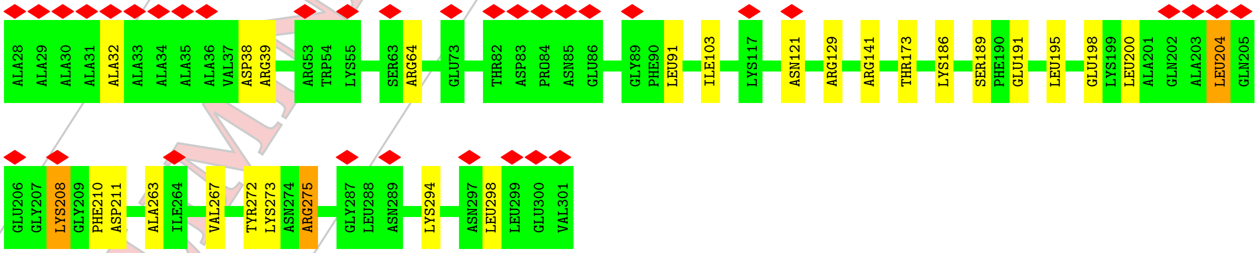

• Molecule 1:

Chain H:

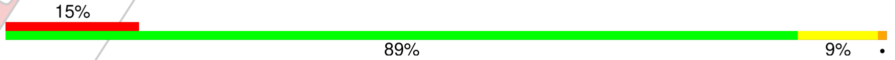

PRELIMINARY VALIDATION REPORT

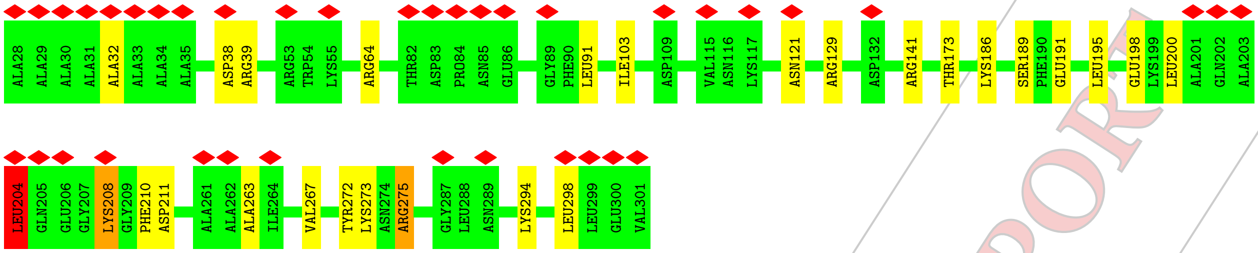

• Molecule 1:

Chain I:

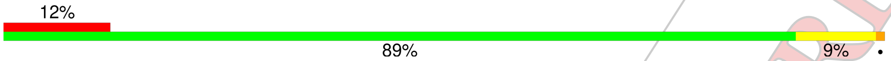

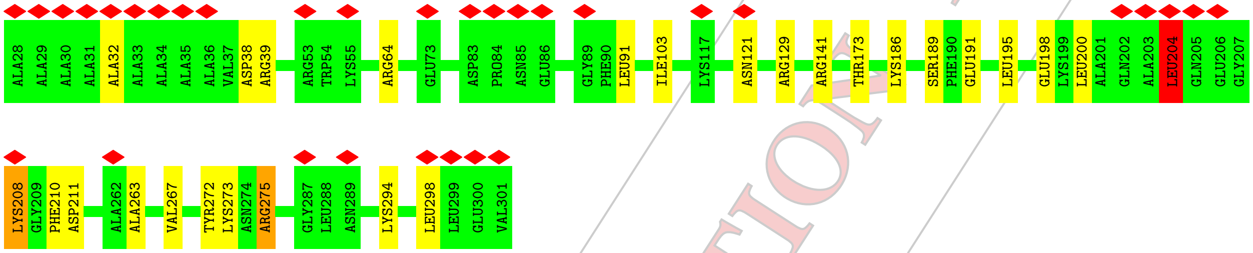

• Molecule 1:

Chain J:

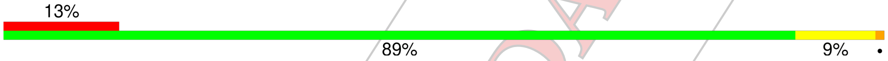

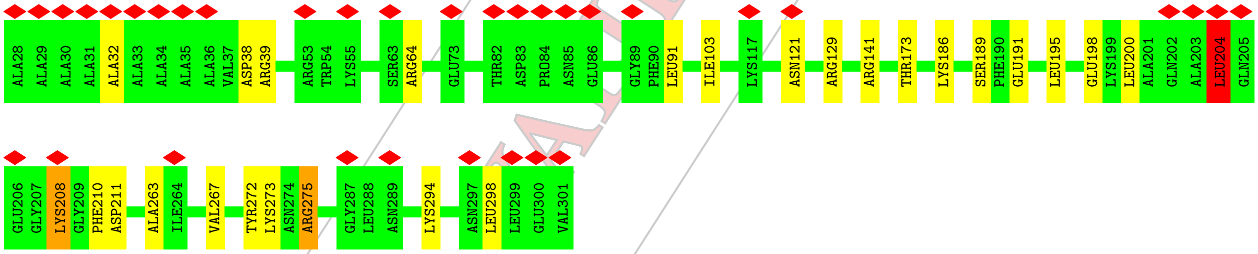

• Molecule 1:

Chain K:

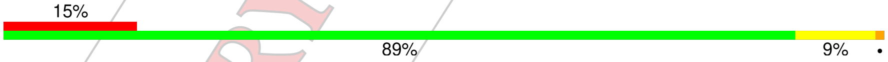

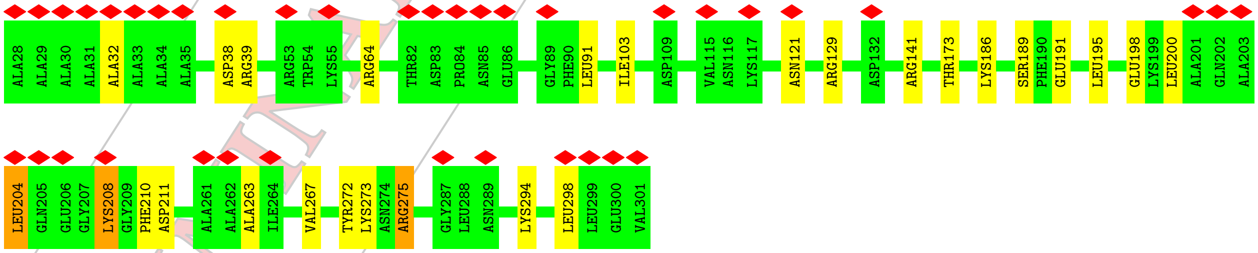

• Molecule 1:

Chain L:

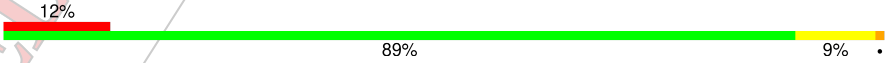

PRELIMINARY VALIDATION REPORT

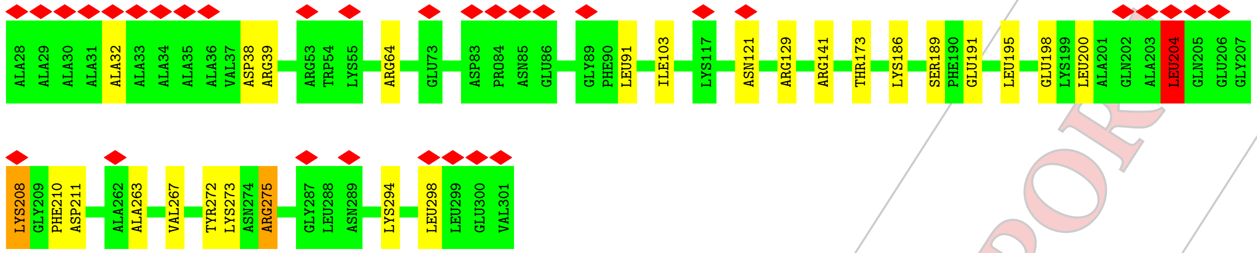

PRELIMINARY VALIDATION REPORT

4 Experimental information (i)

Property	Value	Source
EM reconstruction method	SINGLE PARTICLE	Depositor
Imposed symmetry	POINT, Not provided	
Number of particles used	Not provided	
Resolution determination method	Not provided	
CTF correction method	Not provided	
Microscope	Not provided	
Voltage (kV)	Not provided	
Electron dose ($e^-/\text{Å}^2$)	Not provided	
Minimum defocus (nm)	Not provided	
Maximum defocus (nm)	Not provided	
Magnification	Not provided	
Image detector	Not provided	
Maximum map value	0.200	Depositor
Minimum map value	-0.105	Depositor
Average map value	0.000	Depositor
Map value standard deviation	0.005	Depositor
Recommended contour level	0.023	Depositor
Map size (Å)	321.00003, 321.00003, 321.00003	wwPDB
Map dimensions	300, 300, 300	wwPDB
Map angles (°)	90.0, 90.0, 90.0	wwPDB
Pixel spacing (Å)	1.07, 1.07, 1.07	Depositor

PRELIMINARY VALIDATION REPORT

5 Model quality (i)

5.1 Standard geometry (i)

The Z score for a bond length (or angle) is the number of standard deviations the observed value is removed from the expected value. A bond length (or angle) with $|Z| > 5$ is considered an outlier worth inspection. RMSZ is the root-mean-square of all Z scores of the bond lengths (or angles).

Mol	Chain	Bond lengths		Bond angles	
		RMSZ	#$\|Z\|$ >5	RMSZ	#$\|Z\|$ >5
1	A	0.67	0/2070	1.04	5/2804 (0.2%)
1	B	0.67	0/2070	1.04	5/2804 (0.2%)
1	C	0.67	0/2070	1.04	5/2804 (0.2%)
1	D	0.67	0/2070	1.04	5/2804 (0.2%)
1	E	0.67	0/2070	1.04	5/2804 (0.2%)
1	F	0.67	0/2070	1.04	5/2804 (0.2%)
1	G	0.67	0/2070	1.04	5/2804 (0.2%)
1	H	0.67	0/2070	1.04	5/2804 (0.2%)
1	I	0.67	0/2070	1.04	5/2804 (0.2%)
1	J	0.67	0/2070	1.04	5/2804 (0.2%)
1	K	0.67	0/2070	1.04	5/2804 (0.2%)
1	L	0.67	0/2070	1.04	5/2804 (0.2%)
All	All	0.67	0/24840	1.04	60/33648 (0.2%)

Chiral center outliers are detected by calculating the chiral volume of a chiral center and verifying if the center is modelled as a planar moiety or with the opposite hand.A planarity outlier is detected by checking planarity of atoms in a peptide group, atoms in a mainchain group or atoms of a sidechain that are expected to be planar.

Mol	Chain	#Chirality outliers	#Planarity outliers
1	A	0	1
1	B	0	1
1	C	0	1
1	D	0	1
1	E	0	1
1	F	0	1
1	G	0	1
1	H	0	1
1	I	0	1
1	J	0	1
1	K	0	1
1	L	0	1
All	All	0	12

PRELIMINARY VALIDATION REPORT

There are no bond length outliers.

All (60) bond angle outliers are listed below:

Mol	Chain	Res	Type	Atoms	Z	Observed(^{o})	Ideal(^{o})
1	B	204	LEU	CB-CG-CD1	14.59	135.80	111.00
1	C	204	LEU	CB-CG-CD1	14.59	135.80	111.00
1	E	204	LEU	CB-CG-CD1	14.59	135.80	111.00
1	F	204	LEU	CB-CG-CD1	14.59	135.80	111.00
1	H	204	LEU	CB-CG-CD1	14.59	135.80	111.00
1	I	204	LEU	CB-CG-CD1	14.59	135.80	111.00
1	K	204	LEU	CB-CG-CD1	14.59	135.80	111.00
1	L	204	LEU	CB-CG-CD1	14.59	135.80	111.00
1	A	204	LEU	CB-CG-CD1	14.58	135.78	111.00
1	D	204	LEU	CB-CG-CD1	14.58	135.78	111.00
1	G	204	LEU	CB-CG-CD1	14.58	135.78	111.00
1	J	204	LEU	CB-CG-CD1	14.58	135.78	111.00
1	C	275	ARG	NE-CZ-NH2	9.04	124.82	120.30
1	F	275	ARG	NE-CZ-NH2	9.04	124.82	120.30
1	I	275	ARG	NE-CZ-NH2	9.04	124.82	120.30
1	L	275	ARG	NE-CZ-NH2	9.04	124.82	120.30
1	A	275	ARG	NE-CZ-NH2	8.99	124.80	120.30
1	D	275	ARG	NE-CZ-NH2	8.99	124.80	120.30
1	G	275	ARG	NE-CZ-NH2	8.99	124.80	120.30
1	J	275	ARG	NE-CZ-NH2	8.99	124.80	120.30
1	B	275	ARG	NE-CZ-NH2	8.97	124.79	120.30
1	E	275	ARG	NE-CZ-NH2	8.97	124.79	120.30
1	H	275	ARG	NE-CZ-NH2	8.97	124.79	120.30
1	K	275	ARG	NE-CZ-NH2	8.97	124.79	120.30
1	B	39	ARG	NE-CZ-NH1	6.96	123.78	120.30
1	E	39	ARG	NE-CZ-NH1	6.96	123.78	120.30
1	H	39	ARG	NE-CZ-NH1	6.96	123.78	120.30
1	K	39	ARG	NE-CZ-NH1	6.96	123.78	120.30
1	A	39	ARG	NE-CZ-NH1	6.96	123.78	120.30
1	D	39	ARG	NE-CZ-NH1	6.96	123.78	120.30
1	G	39	ARG	NE-CZ-NH1	6.96	123.78	120.30
1	J	39	ARG	NE-CZ-NH1	6.96	123.78	120.30
1	C	39	ARG	NE-CZ-NH1	6.96	123.78	120.30
1	F	39	ARG	NE-CZ-NH1	6.96	123.78	120.30
1	I	39	ARG	NE-CZ-NH1	6.96	123.78	120.30
1	L	39	ARG	NE-CZ-NH1	6.96	123.78	120.30
1	A	129	ARG	NE-CZ-NH1	6.51	123.56	120.30
1	D	129	ARG	NE-CZ-NH1	6.51	123.56	120.30
1	G	129	ARG	NE-CZ-NH1	6.51	123.56	120.30
1	J	129	ARG	NE-CZ-NH1	6.51	123.56	120.30

Continued on next page...

PRELIMINARY VALIDATION REPORT

Continued from previous page...

Mol	Chain	Res	Type	Atoms	Z	Observed(^{o})	Ideal(^{o})
1	B	129	ARG	NE-CZ-NH1	6.51	123.56	120.30
1	E	129	ARG	NE-CZ-NH1	6.51	123.56	120.30
1	H	129	ARG	NE-CZ-NH1	6.51	123.56	120.30
1	K	129	ARG	NE-CZ-NH1	6.51	123.56	120.30
1	C	129	ARG	NE-CZ-NH1	6.48	123.54	120.30
1	F	129	ARG	NE-CZ-NH1	6.48	123.54	120.30
1	I	129	ARG	NE-CZ-NH1	6.48	123.54	120.30
1	L	129	ARG	NE-CZ-NH1	6.48	123.54	120.30
1	B	64	ARG	NE-CZ-NH1	6.22	123.41	120.30
1	E	64	ARG	NE-CZ-NH1	6.22	123.41	120.30
1	H	64	ARG	NE-CZ-NH1	6.22	123.41	120.30
1	K	64	ARG	NE-CZ-NH1	6.22	123.41	120.30
1	A	64	ARG	NE-CZ-NH1	6.19	123.39	120.30
1	D	64	ARG	NE-CZ-NH1	6.19	123.39	120.30
1	G	64	ARG	NE-CZ-NH1	6.19	123.39	120.30
1	J	64	ARG	NE-CZ-NH1	6.19	123.39	120.30
1	C	64	ARG	NE-CZ-NH1	6.14	123.37	120.30
1	F	64	ARG	NE-CZ-NH1	6.14	123.37	120.30
1	I	64	ARG	NE-CZ-NH1	6.14	123.37	120.30
1	L	64	ARG	NE-CZ-NH1	6.14	123.37	120.30

There are no chirality outliers.

All (12) planarity outliers are listed below:

Mol	Chain	Res	Type	Group
1	A	275	ARG	Sidechain
1	B	275	ARG	Sidechain
1	C	275	ARG	Sidechain
1	D	275	ARG	Sidechain
1	E	275	ARG	Sidechain
1	F	275	ARG	Sidechain
1	G	275	ARG	Sidechain
1	H	275	ARG	Sidechain
1	I	275	ARG	Sidechain
1	J	275	ARG	Sidechain
1	K	275	ARG	Sidechain
1	L	275	ARG	Sidechain

PRELIMINARY VALIDATION REPORT

5.2 Too-close contacts (i)

In the following table, the Non-H and H(model) columns list the number of non-hydrogen atoms and hydrogen atoms in the chain respectively. The H(added) column lists the number of hydrogen atoms added and optimized by MolProbity. The Clashes column lists the number of clashes within the asymmetric unit, whereas Symm-Clashes lists symmetry-related clashes.

Mol	Chain	Non-H	H(model)	H(added)	Clashes	Symm-Clashes
1	A	2028	0	1994	35	0
1	B	2028	0	1994	34	0
1	C	2028	0	1994	34	0
1	D	2028	0	1994	34	0
1	E	2028	0	1994	34	0
1	F	2028	0	1994	34	0
1	G	2028	0	1994	34	0
1	H	2028	0	1994	36	0
1	I	2028	0	1994	38	0
1	J	2028	0	1994	37	0
1	K	2028	0	1994	36	0
1	L	2028	0	1994	38	0
All	All	24336	0	23928	229	0

The all-atom clashscore is defined as the number of clashes found per 1000 atoms (including hydrogen atoms). The all-atom clashscore for this structure is 5.

All (229) close contacts within the same asymmetric unit are listed below, sorted by their clash magnitude.

Atom-1	Atom-2	Interatomic distance (Å)	Clash overlap (Å)
1:H:298:LEU:HD12	1:I:273:LYS:CE	1.63	1.29
1:E:298:LEU:HD12	1:F:273:LYS:CE	1.63	1.29
1:J:298:LEU:HD12	1:K:273:LYS:CE	1.63	1.29
1:C:298:LEU:HD12	1:D:273:LYS:CE	1.63	1.28
1:F:298:LEU:HD12	1:G:273:LYS:CE	1.63	1.28
1:G:298:LEU:HD12	1:H:273:LYS:CE	1.63	1.28
1:K:298:LEU:HD12	1:L:273:LYS:CE	1.63	1.28
1:A:273:LYS:CE	1:L:298:LEU:HD12	1.63	1.27
1:A:298:LEU:HD12	1:B:273:LYS:CE	1.63	1.27
1:D:298:LEU:HD12	1:E:273:LYS:CE	1.63	1.27
1:I:298:LEU:HD12	1:J:273:LYS:CE	1.63	1.27
1:B:298:LEU:HD12	1:C:273:LYS:CE	1.63	1.26
1:H:298:LEU:CD1	1:I:273:LYS:HE2	1.70	1.22
1:F:298:LEU:CD1	1:G:273:LYS:HE2	1.70	1.22
1:K:298:LEU:CD1	1:L:273:LYS:HE2	1.70	1.21

Continued on next page...

PRELIMINARY VALIDATION REPORT

Continued from previous page...

Atom-1	Atom-2	Interatomic distance (Å)	Clash overlap (Å)
1:J:298:LEU:CD1	1:K:273:LYS:HE2	1.70	1.21
1:E:298:LEU:CD1	1:F:273:LYS:HE2	1.70	1.21
1:I:298:LEU:CD1	1:J:273:LYS:HE2	1.70	1.21
1:C:298:LEU:CD1	1:D:273:LYS:HE2	1.70	1.21
1:D:298:LEU:CD1	1:E:273:LYS:HE2	1.70	1.21
1:A:298:LEU:CD1	1:B:273:LYS:HE2	1.70	1.20
1:G:298:LEU:CD1	1:H:273:LYS:HE2	1.70	1.19
1:A:273:LYS:HE2	1:L:298:LEU:CD1	1.70	1.19
1:B:298:LEU:CD1	1:C:273:LYS:HE2	1.70	1.19
1:J:298:LEU:HD12	1:K:273:LYS:HE2	0.85	0.84
1:G:298:LEU:HD12	1:H:273:LYS:HE2	0.85	0.83
1:D:298:LEU:HD12	1:E:273:LYS:HE2	0.85	0.83
1:K:298:LEU:HD12	1:L:273:LYS:HE2	0.84	0.82
1:H:298:LEU:HD12	1:I:273:LYS:HE2	0.84	0.81
1:A:298:LEU:HD12	1:B:273:LYS:HE2	0.85	0.81
1:A:273:LYS:HE2	1:L:298:LEU:HD12	0.84	0.81
1:B:298:LEU:HD12	1:C:273:LYS:HE2	0.84	0.81
1:E:298:LEU:HD12	1:F:273:LYS:HE2	0.84	0.81
1:C:298:LEU:HD12	1:D:273:LYS:HE2	0.84	0.80
1:F:298:LEU:HD12	1:G:273:LYS:HE2	0.84	0.79
1:I:298:LEU:HD12	1:J:273:LYS:HE2	0.84	0.79
1:G:186:LYS:NZ	1:H:191:GLU:OE1	2.14	0.79
1:C:186:LYS:NZ	1:D:191:GLU:OE1	2.14	0.79
1:J:186:LYS:NZ	1:K:191:GLU:OE1	2.14	0.78
1:F:186:LYS:NZ	1:G:191:GLU:OE1	2.14	0.78
1:I:186:LYS:NZ	1:J:191:GLU:OE1	2.14	0.77
1:D:186:LYS:NZ	1:E:191:GLU:OE1	2.14	0.76
1:A:191:GLU:OE1	1:L:186:LYS:NZ	2.14	0.76
1:A:273:LYS:HD3	1:L:298:LEU:HB3	1.68	0.76
1:A:298:LEU:HB3	1:B:273:LYS:HD3	1.68	0.76
1:E:298:LEU:HB3	1:F:273:LYS:HD3	1.68	0.76
1:F:298:LEU:HB3	1:G:273:LYS:HD3	1.68	0.76
1:K:298:LEU:HB3	1:L:273:LYS:HD3	1.68	0.76
1:D:298:LEU:HB3	1:E:273:LYS:HD3	1.68	0.76
1:B:298:LEU:HB3	1:C:273:LYS:HD3	1.68	0.75
1:C:298:LEU:HB3	1:D:273:LYS:HD3	1.68	0.75
1:J:298:LEU:HB3	1:K:273:LYS:HD3	1.68	0.75
1:A:186:LYS:NZ	1:B:191:GLU:OE1	2.14	0.75
1:G:298:LEU:HB3	1:H:273:LYS:HD3	1.68	0.75
1:E:186:LYS:NZ	1:F:191:GLU:OE1	2.14	0.75
1:H:298:LEU:HB3	1:I:273:LYS:HD3	1.68	0.75

Continued on next page...

PRELIMINARY VALIDATION REPORT

Continued from previous page...

Atom-1	Atom-2	Interatomic distance (Å)	Clash overlap (Å)
1:I:298:LEU:HB3	1:J:273:LYS:HD3	1.68	0.75
1:D:173:THR:OG1	1:E:198:GLU:OE2	2.05	0.75
1:E:173:THR:OG1	1:F:198:GLU:OE2	2.05	0.75
1:K:186:LYS:NZ	1:L:191:GLU:OE1	2.14	0.74
1:B:173:THR:OG1	1:C:198:GLU:OE2	2.05	0.74
1:A:173:THR:OG1	1:B:198:GLU:OE2	2.05	0.74
1:G:173:THR:OG1	1:H:198:GLU:OE2	2.05	0.73
1:H:173:THR:OG1	1:I:198:GLU:OE2	2.05	0.73
1:H:186:LYS:NZ	1:I:191:GLU:OE1	2.14	0.73
1:B:186:LYS:NZ	1:C:191:GLU:OE1	2.14	0.73
1:C:173:THR:OG1	1:D:198:GLU:OE2	2.05	0.73
1:F:173:THR:OG1	1:G:198:GLU:OE2	2.05	0.73
1:A:198:GLU:OE2	1:L:173:THR:OG1	2.05	0.73
1:I:173:THR:OG1	1:J:198:GLU:OE2	2.05	0.72
1:B:208:LYS:NZ	1:C:211:ASP:OD2	2.21	0.72
1:J:173:THR:OG1	1:K:198:GLU:OE2	2.05	0.71
1:K:173:THR:OG1	1:L:198:GLU:OE2	2.05	0.71
1:G:208:LYS:NZ	1:H:211:ASP:OD2	2.21	0.70
1:H:208:LYS:NZ	1:I:211:ASP:OD2	2.21	0.70
1:E:208:LYS:NZ	1:F:211:ASP:OD2	2.21	0.69
1:F:208:LYS:NZ	1:G:211:ASP:OD2	2.21	0.69
1:G:263:ALA:O	1:G:267:VAL:HG23	1.93	0.69
1:I:208:LYS:NZ	1:J:211:ASP:OD2	2.21	0.69
1:F:263:ALA:O	1:F:267:VAL:HG23	1.93	0.69
1:A:211:ASP:OD2	1:L:208:LYS:NZ	2.21	0.69
1:H:263:ALA:O	1:H:267:VAL:HG23	1.93	0.69
1:D:263:ALA:O	1:D:267:VAL:HG23	1.93	0.69
1:I:263:ALA:O	1:I:267:VAL:HG23	1.93	0.69
1:A:263:ALA:O	1:A:267:VAL:HG23	1.93	0.68
1:B:263:ALA:O	1:B:267:VAL:HG23	1.93	0.68
1:E:263:ALA:O	1:E:267:VAL:HG23	1.93	0.68
1:J:263:ALA:O	1:J:267:VAL:HG23	1.93	0.68
1:L:263:ALA:O	1:L:267:VAL:HG23	1.93	0.68
1:C:263:ALA:O	1:C:267:VAL:HG23	1.93	0.68
1:K:263:ALA:O	1:K:267:VAL:HG23	1.93	0.68
1:J:208:LYS:NZ	1:K:211:ASP:OD2	2.21	0.68
1:H:208:LYS:HE3	1:I:200:LEU:HD13	1.77	0.67
1:I:208:LYS:HE3	1:J:200:LEU:HD13	1.77	0.67
1:J:208:LYS:HE3	1:K:200:LEU:HD13	1.77	0.67
1:K:208:LYS:HE3	1:L:200:LEU:HD13	1.77	0.67
1:G:208:LYS:HE3	1:H:200:LEU:HD13	1.77	0.67

Continued on next page...

PRELIMINARY VALIDATION REPORT

Continued from previous page...

Atom-1	Atom-2	Interatomic distance (Å)	Clash overlap (Å)
1:K:208:LYS:NZ	1:L:211:ASP:OD2	2.21	0.67
1:A:200:LEU:HD13	1:L:208:LYS:HE3	1.77	0.67
1:D:208:LYS:NZ	1:E:211:ASP:OD2	2.21	0.67
1:A:208:LYS:HE3	1:B:200:LEU:HD13	1.77	0.66
1:F:208:LYS:HE3	1:G:200:LEU:HD13	1.77	0.66
1:B:208:LYS:HE3	1:C:200:LEU:HD13	1.77	0.66
1:H:189:SER:O	1:I:32:ALA:HB2	1.96	0.66
1:K:189:SER:O	1:L:32:ALA:HB2	1.96	0.66
1:F:189:SER:O	1:G:32:ALA:HB2	1.96	0.66
1:I:189:SER:O	1:J:32:ALA:HB2	1.96	0.66
1:D:189:SER:O	1:E:32:ALA:HB2	1.96	0.66
1:G:189:SER:O	1:H:32:ALA:HB2	1.96	0.65
1:B:189:SER:O	1:C:32:ALA:HB2	1.96	0.65
1:C:208:LYS:HE3	1:D:200:LEU:HD13	1.77	0.65
1:J:189:SER:O	1:K:32:ALA:HB2	1.96	0.65
1:A:32:ALA:HB2	1:L:189:SER:O	1.96	0.65
1:A:189:SER:O	1:B:32:ALA:HB2	1.96	0.65
1:D:208:LYS:HE3	1:E:200:LEU:HD13	1.77	0.65
1:E:208:LYS:HE3	1:F:200:LEU:HD13	1.77	0.65
1:C:208:LYS:NZ	1:D:211:ASP:OD2	2.21	0.65
1:C:189:SER:O	1:D:32:ALA:HB2	1.96	0.65
1:K:38:ASP:HB2	1:L:141:ARG:HH12	1.62	0.65
1:B:38:ASP:HB2	1:C:141:ARG:HH12	1.62	0.64
1:D:38:ASP:HB2	1:E:141:ARG:HH12	1.62	0.64
1:A:141:ARG:HH12	1:L:38:ASP:HB2	1.62	0.64
1:C:38:ASP:HB2	1:D:141:ARG:HH12	1.62	0.64
1:A:38:ASP:HB2	1:B:141:ARG:HH12	1.62	0.64
1:E:189:SER:O	1:F:32:ALA:HB2	1.96	0.64
1:F:38:ASP:HB2	1:G:141:ARG:HH12	1.62	0.64
1:H:38:ASP:HB2	1:I:141:ARG:HH12	1.62	0.64
1:I:38:ASP:HB2	1:J:141:ARG:HH12	1.62	0.64
1:J:38:ASP:HB2	1:K:141:ARG:HH12	1.62	0.64
1:E:38:ASP:HB2	1:F:141:ARG:HH12	1.62	0.63
1:G:38:ASP:HB2	1:H:141:ARG:HH12	1.62	0.63
1:D:210:PHE:CE2	1:E:195:LEU:HD11	2.34	0.63
1:K:210:PHE:CE2	1:L:195:LEU:HD11	2.34	0.63
1:C:210:PHE:CE2	1:D:195:LEU:HD11	2.34	0.63
1:E:210:PHE:CE2	1:F:195:LEU:HD11	2.34	0.63
1:J:210:PHE:CE2	1:K:195:LEU:HD11	2.34	0.63
1:A:208:LYS:NZ	1:B:211:ASP:OD2	2.21	0.62
1:B:210:PHE:CE2	1:C:195:LEU:HD11	2.34	0.62

Continued on next page...

PRELIMINARY VALIDATION REPORT

Continued from previous page...

Atom-1	Atom-2	Interatomic distance (Å)	Clash overlap (Å)
1:F:210:PHE:CE2	1:G:195:LEU:HD11	2.34	0.62
1:A:195:LEU:HD11	1:L:210:PHE:CE2	2.34	0.62
1:A:210:PHE:CE2	1:B:195:LEU:HD11	2.34	0.62
1:I:210:PHE:CE2	1:J:195:LEU:HD11	2.34	0.62
1:G:210:PHE:CE2	1:H:195:LEU:HD11	2.34	0.62
1:H:210:PHE:CE2	1:I:195:LEU:HD11	2.34	0.62
1:F:298:LEU:HD12	1:G:273:LYS:HE3	1.78	0.59
1:I:298:LEU:HD12	1:J:273:LYS:HE3	1.78	0.58
1:C:38:ASP:CB	1:D:141:ARG:HH12	2.18	0.57
1:D:38:ASP:CB	1:E:141:ARG:HH12	2.18	0.57
1:A:141:ARG:HH12	1:L:38:ASP:CB	2.18	0.57
1:B:38:ASP:CB	1:C:141:ARG:HH12	2.18	0.57
1:E:38:ASP:CB	1:F:141:ARG:HH12	2.18	0.57
1:F:38:ASP:CB	1:G:141:ARG:HH12	2.18	0.57
1:A:38:ASP:CB	1:B:141:ARG:HH12	2.18	0.57
1:G:38:ASP:CB	1:H:141:ARG:HH12	2.18	0.56
1:C:298:LEU:HD12	1:D:273:LYS:HE3	1.78	0.56
1:H:38:ASP:CB	1:I:141:ARG:HH12	2.18	0.56
1:J:38:ASP:CB	1:K:141:ARG:HH12	2.18	0.56
1:K:38:ASP:CB	1:L:141:ARG:HH12	2.18	0.56
1:I:38:ASP:CB	1:J:141:ARG:HH12	2.18	0.56
1:A:103:ILE:CD1	1:L:91:LEU:HD22	2.37	0.55
1:C:91:LEU:HD22	1:D:103:ILE:CD1	2.37	0.55
1:D:91:LEU:HD22	1:E:103:ILE:CD1	2.37	0.55
1:E:91:LEU:HD22	1:F:103:ILE:CD1	2.37	0.55
1:F:91:LEU:HD22	1:G:103:ILE:CD1	2.37	0.55
1:K:91:LEU:HD22	1:L:103:ILE:CD1	2.37	0.55
1:E:38:ASP:HB2	1:F:141:ARG:NH1	2.22	0.55
1:G:91:LEU:HD22	1:H:103:ILE:CD1	2.37	0.55
1:A:273:LYS:HE3	1:L:298:LEU:HD12	1.78	0.55
1:G:38:ASP:HB2	1:H:141:ARG:NH1	2.22	0.55
1:H:91:LEU:HD22	1:I:103:ILE:CD1	2.37	0.55
1:I:91:LEU:HD22	1:J:103:ILE:CD1	2.37	0.55
1:J:91:LEU:HD22	1:K:103:ILE:CD1	2.37	0.55
1:F:38:ASP:HB2	1:G:141:ARG:NH1	2.22	0.55
1:B:91:LEU:HD22	1:C:103:ILE:CD1	2.37	0.55
1:A:141:ARG:NH1	1:L:38:ASP:HB2	2.22	0.55
1:C:38:ASP:HB2	1:D:141:ARG:NH1	2.22	0.55
1:A:91:LEU:HD22	1:B:103:ILE:CD1	2.37	0.54
1:H:38:ASP:HB2	1:I:141:ARG:NH1	2.22	0.54
1:J:38:ASP:HB2	1:K:141:ARG:NH1	2.22	0.54

Continued on next page...

PRELIMINARY VALIDATION REPORT

Continued from previous page...

Atom-1	Atom-2	Interatomic distance (Å)	Clash overlap (Å)
1:D:38:ASP:HB2	1:E:141:ARG:NH1	2.22	0.54
1:H:298:LEU:HD12	1:I:273:LYS:HE3	1.79	0.54
1:B:38:ASP:HB2	1:C:141:ARG:NH1	2.22	0.54
1:I:38:ASP:HB2	1:J:141:ARG:NH1	2.22	0.54
1:A:38:ASP:HB2	1:B:141:ARG:NH1	2.22	0.53
1:E:298:LEU:HD12	1:F:273:LYS:HE3	1.79	0.53
1:K:38:ASP:HB2	1:L:141:ARG:NH1	2.22	0.53
1:K:298:LEU:HD12	1:L:273:LYS:HE3	1.79	0.51
1:B:298:LEU:HD12	1:C:273:LYS:HE3	1.79	0.50
1:G:298:LEU:HD12	1:H:273:LYS:HE3	1.78	0.48
1:D:298:LEU:HD12	1:E:273:LYS:HE3	1.78	0.46
1:J:298:LEU:HD12	1:K:273:LYS:HE3	1.78	0.46
1:A:298:LEU:HD12	1:B:273:LYS:HE3	1.78	0.45
1:L:204:LEU:H	1:L:204:LEU:HG	1.78	0.44
1:H:272:TYR:CD2	1:H:294:LYS:HE3	2.53	0.44
1:I:272:TYR:CD2	1:I:294:LYS:HE3	2.53	0.44
1:J:272:TYR:CD2	1:J:294:LYS:HE3	2.53	0.44
1:K:272:TYR:CD2	1:K:294:LYS:HE3	2.53	0.44
1:G:272:TYR:CD2	1:G:294:LYS:HE3	2.53	0.44
1:L:272:TYR:CD2	1:L:294:LYS:HE3	2.53	0.44
1:F:272:TYR:CD2	1:F:294:LYS:HE3	2.53	0.44
1:A:272:TYR:CD2	1:A:294:LYS:HE3	2.53	0.43
1:C:272:TYR:CD2	1:C:294:LYS:HE3	2.53	0.43
1:D:272:TYR:CD2	1:D:294:LYS:HE3	2.53	0.43
1:B:272:TYR:CD2	1:B:294:LYS:HE3	2.53	0.43
1:E:272:TYR:CD2	1:E:294:LYS:HE3	2.53	0.43
1:J:204:LEU:H	1:J:204:LEU:HG	1.78	0.42
1:D:204:LEU:H	1:D:204:LEU:HG	1.78	0.42
1:C:204:LEU:H	1:C:204:LEU:HG	1.78	0.42
1:A:200:LEU:CD1	1:L:208:LYS:HE3	2.49	0.42
1:E:204:LEU:H	1:E:204:LEU:HG	1.78	0.41
1:I:208:LYS:HE3	1:J:200:LEU:CD1	2.49	0.41
1:B:204:LEU:H	1:B:204:LEU:HG	1.78	0.41
1:F:204:LEU:H	1:F:204:LEU:HG	1.78	0.41
1:G:210:PHE:CE2	1:H:195:LEU:CD1	3.04	0.41
1:H:204:LEU:H	1:H:204:LEU:HG	1.78	0.41
1:H:210:PHE:CZ	1:I:195:LEU:HD11	2.56	0.41
1:A:204:LEU:H	1:A:204:LEU:HG	1.78	0.41
1:K:210:PHE:CZ	1:L:195:LEU:HD11	2.56	0.41
1:A:195:LEU:CD1	1:L:210:PHE:CE2	3.04	0.40
1:D:210:PHE:CE2	1:E:195:LEU:CD1	3.04	0.40

Continued on next page...

PRELIMINARY VALIDATION REPORT

Continued from previous page...

Atom-1	Atom-2	Interatomic distance (Å)	Clash overlap (Å)
1:H:208:LYS:HE3	1:I:200:LEU:CD1	2.49	0.40
1:I:210:PHE:CE2	1:J:195:LEU:CD1	3.04	0.40
1:I:210:PHE:CZ	1:J:195:LEU:HD11	2.56	0.40
1:I:204:LEU:H	1:I:204:LEU:HG	1.78	0.40
1:B:210:PHE:CZ	1:C:195:LEU:HD11	2.56	0.40
1:F:210:PHE:CZ	1:G:195:LEU:HD11	2.56	0.40
1:J:210:PHE:CZ	1:K:195:LEU:HD11	2.56	0.40
1:K:208:LYS:HE3	1:L:200:LEU:CD1	2.49	0.40
1:K:210:PHE:CE2	1:L:195:LEU:CD1	3.04	0.40

There are no symmetry-related clashes.

5.3 Torsion angles (i)

5.3.1 Protein backbone (i)

In the following table, the Percentiles column shows the percent Ramachandran outliers of the chain as a percentile score with respect to all PDB entries followed by that with respect to all EM entries.

The Analysed column shows the number of residues for which the backbone conformation was analysed, and the total number of residues.

Mol	Chain	Analysed	Favoured	Allowed	Outliers	Percentiles	
1	A	252/256 (98%)	248 (98%)	3 (1%)	1 (0%)	34	34
1	B	252/256 (98%)	248 (98%)	3 (1%)	1 (0%)	34	34
1	C	252/256 (98%)	248 (98%)	3 (1%)	1 (0%)	34	34
1	D	252/256 (98%)	248 (98%)	3 (1%)	1 (0%)	34	34
1	E	252/256 (98%)	248 (98%)	3 (1%)	1 (0%)	34	34
1	F	252/256 (98%)	248 (98%)	3 (1%)	1 (0%)	34	34
1	G	252/256 (98%)	248 (98%)	3 (1%)	1 (0%)	34	34
1	H	252/256 (98%)	248 (98%)	3 (1%)	1 (0%)	34	34
1	I	252/256 (98%)	248 (98%)	3 (1%)	1 (0%)	34	34
1	J	252/256 (98%)	248 (98%)	3 (1%)	1 (0%)	34	34
1	K	252/256 (98%)	248 (98%)	3 (1%)	1 (0%)	34	34
1	L	252/256 (98%)	248 (98%)	3 (1%)	1 (0%)	34	34
All	All	3024/3072 (98%)	2976 (98%)	36 (1%)	12 (0%)	38	34

PRELIMINARY VALIDATION REPORT

All (12) Ramachandran outliers are listed below:

Mol	Chain	Res	Type
1	A	121	ASN
1	B	121	ASN
1	C	121	ASN
1	D	121	ASN
1	E	121	ASN
1	F	121	ASN
1	G	121	ASN
1	H	121	ASN
1	I	121	ASN
1	J	121	ASN
1	K	121	ASN
1	L	121	ASN

5.3.2 Protein sidechains (i)

In the following table, the Percentiles column shows the percent sidechain outliers of the chain as a percentile score with respect to all PDB entries followed by that with respect to all EM entries.

The Analysed column shows the number of residues for which the sidechain conformation was analysed, and the total number of residues.

Mol	Chain	Analysed	Rotameric	Outliers	Percentiles	
1	A	215/215 (100%)	213 (99%)	2 (1%)	78	78
1	B	215/215 (100%)	213 (99%)	2 (1%)	78	78
1	C	215/215 (100%)	213 (99%)	2 (1%)	78	78
1	D	215/215 (100%)	213 (99%)	2 (1%)	78	78
1	E	215/215 (100%)	213 (99%)	2 (1%)	78	78
1	F	215/215 (100%)	213 (99%)	2 (1%)	78	78
1	G	215/215 (100%)	213 (99%)	2 (1%)	78	78
1	H	215/215 (100%)	213 (99%)	2 (1%)	78	78
1	I	215/215 (100%)	213 (99%)	2 (1%)	78	78
1	J	215/215 (100%)	213 (99%)	2 (1%)	78	78
1	K	215/215 (100%)	213 (99%)	2 (1%)	78	78
1	L	215/215 (100%)	213 (99%)	2 (1%)	78	78
All	All	2580/2580 (100%)	2556 (99%)	24 (1%)	79	78

PRELIMINARY VALIDATION REPORT

All (24) residues with a non-rotameric sidechain are listed below:

Mol	Chain	Res	Type
1	A	204	LEU
1	A	208	LYS
1	B	204	LEU
1	B	208	LYS
1	C	204	LEU
1	C	208	LYS
1	D	204	LEU
1	D	208	LYS
1	E	204	LEU
1	E	208	LYS
1	F	204	LEU
1	F	208	LYS
1	G	204	LEU
1	G	208	LYS
1	H	204	LEU
1	H	208	LYS
1	I	204	LEU
1	I	208	LYS
1	J	204	LEU
1	J	208	LYS
1	K	204	LEU
1	K	208	LYS
1	L	204	LEU
1	L	208	LYS

Sometimes sidechains can be flipped to improve hydrogen bonding and reduce clashes. There are no such sidechains identified.

5.3.3 RNA (i)

There are no RNA molecules in this entry.

5.4 Non-standard residues in protein, DNA, RNA chains (i)

There are no non-standard protein/DNA/RNA residues in this entry.

5.5 Carbohydrates (i)

There are no monosaccharides in this entry.

PRELIMINARY VALIDATION REPORT

5.6 Ligand geometry (i)

There are no ligands in this entry.

5.7 Other polymers (i)

There are no such residues in this entry.

5.8 Polymer linkage issues (i)

The following chains have linkage breaks:

Mol	Chain	Number of breaks
1	A	1
1	B	1
1	C	1
1	D	1
1	E	1
1	F	1
1	G	1
1	H	1
1	I	1
1	J	1
1	K	1
1	L	1

All chain breaks are listed below:

Model	Chain	Residue-1	Atom-1	Residue-2	Atom-2	Distance (Å)
1	A	242:ALA	C	261:ALA	N	8.12
1	B	242:ALA	C	261:ALA	N	8.12
1	C	242:ALA	C	261:ALA	N	8.12
1	D	242:ALA	C	261:ALA	N	8.12
1	E	242:ALA	C	261:ALA	N	8.12
1	F	242:ALA	C	261:ALA	N	8.12
1	G	242:ALA	C	261:ALA	N	8.12
1	H	242:ALA	C	261:ALA	N	8.12
1	I	242:ALA	C	261:ALA	N	8.12
1	J	242:ALA	C	261:ALA	N	8.12
1	K	242:ALA	C	261:ALA	N	8.12
1	L	242:ALA	C	261:ALA	N	8.12

PRELIMINARY VALIDATION REPORT

6 Map visualisation (i)

This section contains visualisations of the EMDB entry D_1292118365. These allow visual inspection of the internal detail of the map and identification of artifacts.

No raw map or half-maps were deposited for this entry and therefore no images, graphs, etc. pertaining to the raw map can be shown.

6.1 Orthogonal projections (i)

6.1.1 Primary map

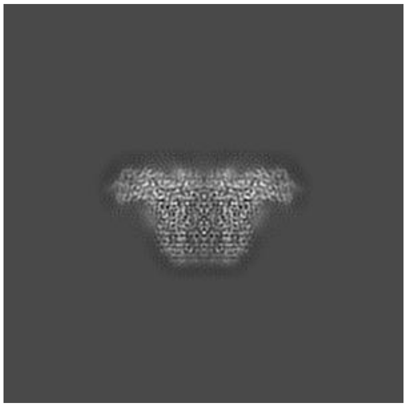
X

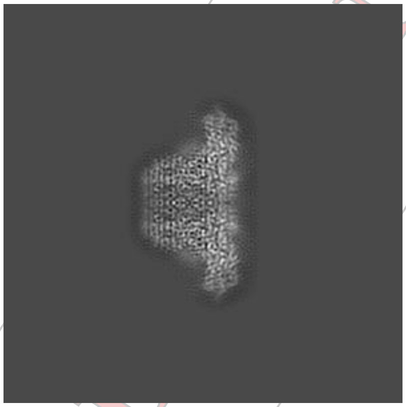
Y

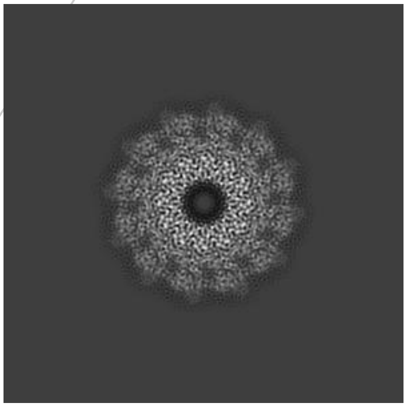
Z

The images above show the map projected in three orthogonal directions.

6.2 Central slices (i)

6.2.1 Primary map

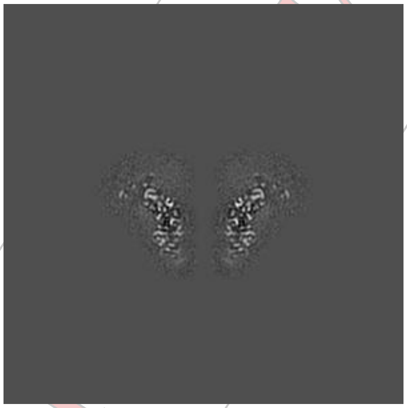
X Index: 150

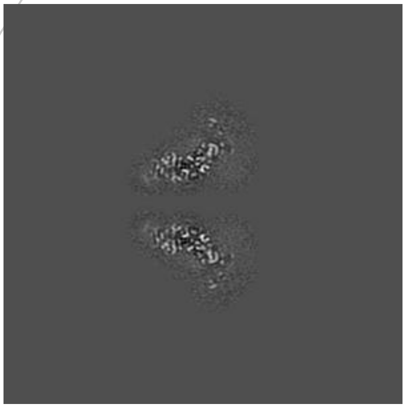
Y Index: 150

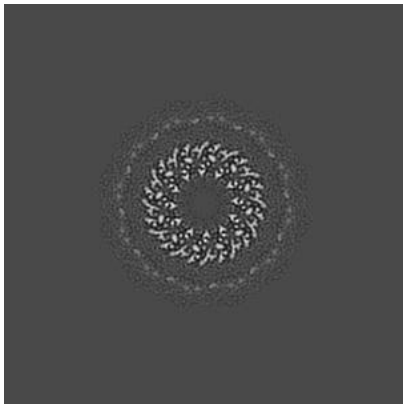
Z Index: 150

PRELIMINARY VALIDATION REPORT

The images above show central slices of the map in three orthogonal directions.

6.3 Largest variance slices (i)

6.3.1 Primary map

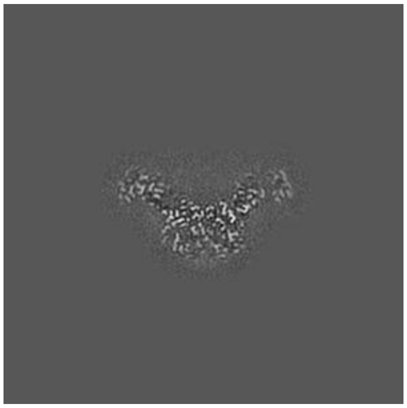
X Index: 170

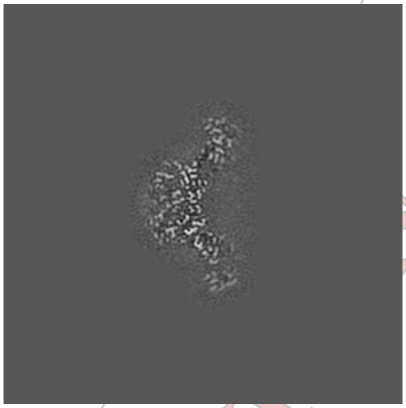
Y Index: 170

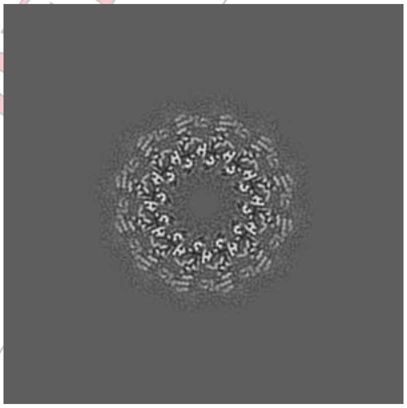
Z Index: 155

The images above show the largest variance slices of the map in three orthogonal directions.

6.4 Orthogonal surface views (i)

6.4.1 Primary map

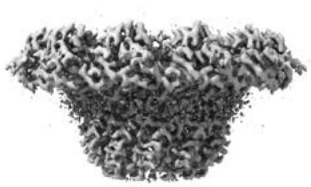
X

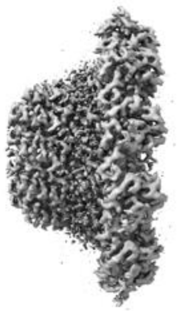
Y

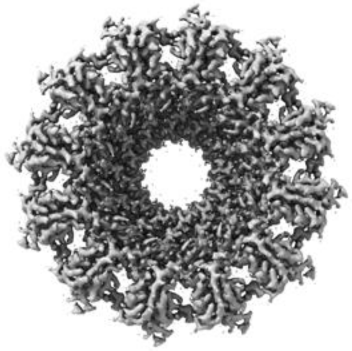
Z

The images above show the 3D surface view of the map at the recommended contour level 0.023. These images, in conjunction with the slice images, may facilitate assessment of whether an appropriate contour level has been provided.

PRELIMINARY VALIDATION REPORT

6.5 Mask visualisation (i)

This section was not generated. No masks/segmentation were deposited.

PRELIMINARY VALIDATION REPORT

7 Map analysis (i)

This section contains the results of statistical analysis of the map.

7.1 Map-value distribution (i)

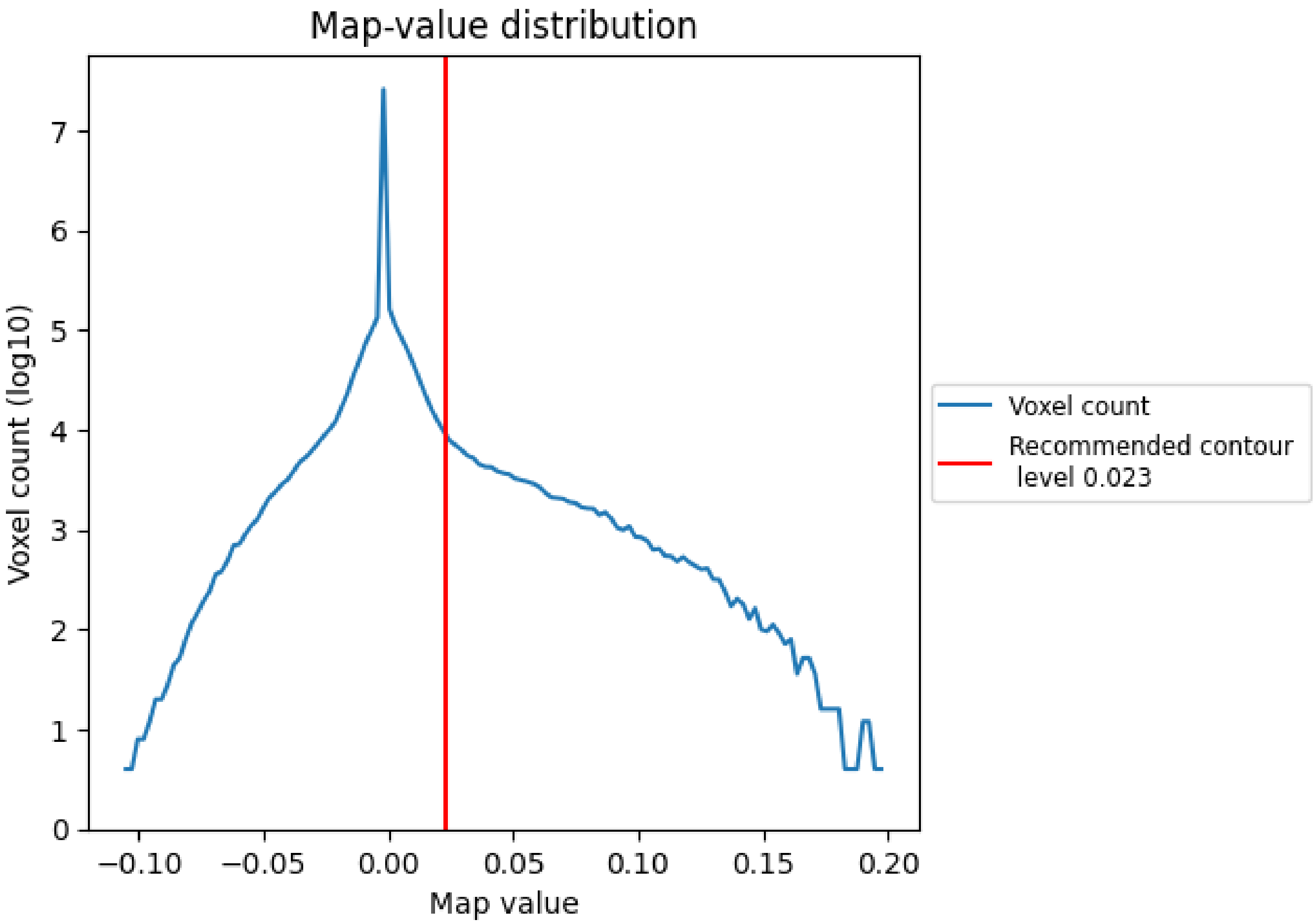

The map-value distribution is plotted in 128 intervals along the x-axis. The y-axis is logarithmic. A spike in this graph at zero usually indicates that the volume has been masked.

PRELIMINARY REPORT

7.2 Volume estimate (i)

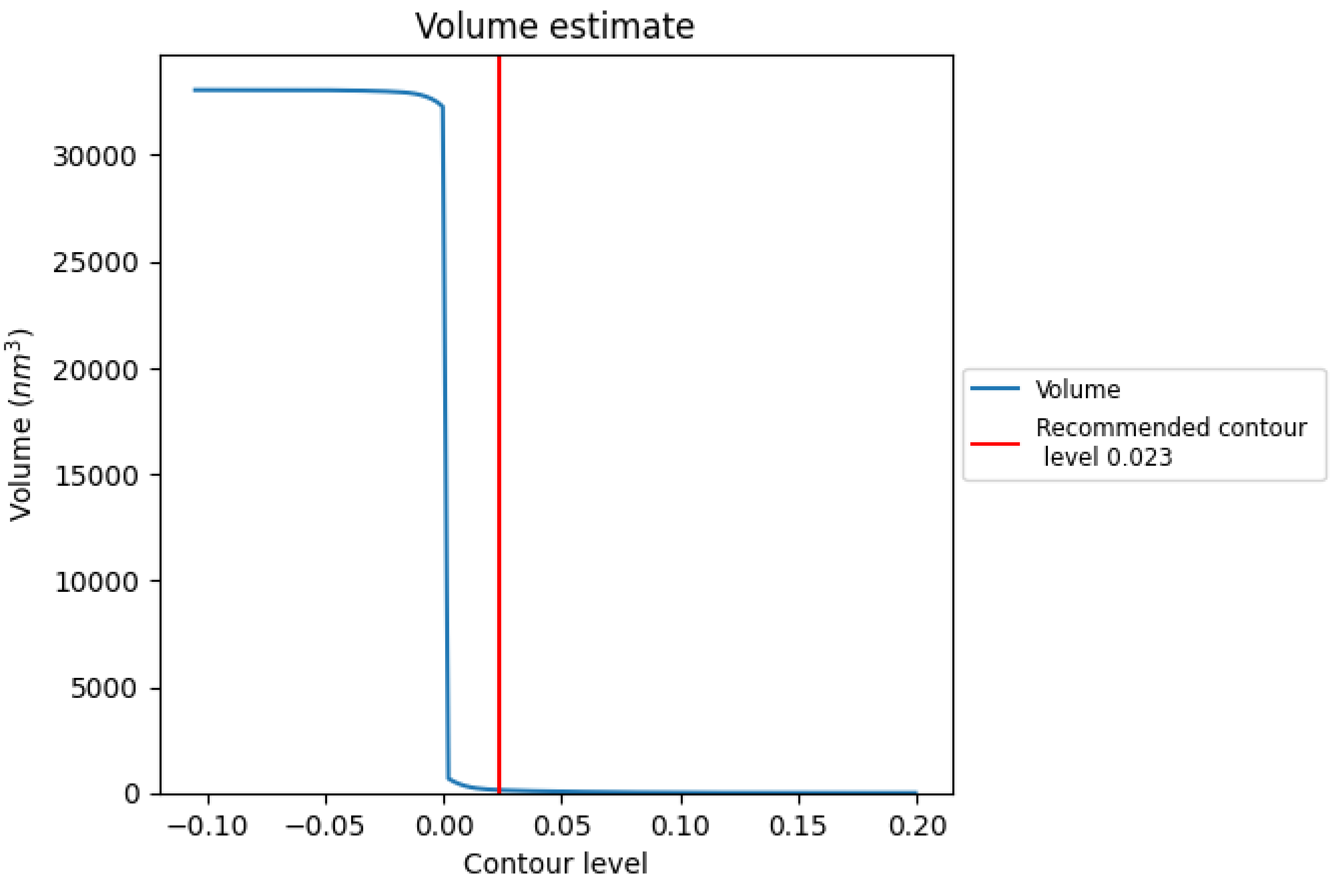

The volume at the recommended contour level is 137 nm^3; this corresponds to an approximate mass of 124 kDa.

The volume estimate graph shows how the enclosed volume varies with the contour level. The recommended contour level is shown as a vertical line and the intersection between the line and the curve gives the volume of the enclosed surface at the given level.

PRELIMINARY VA

7.3 Rotationally averaged power spectrum (i)

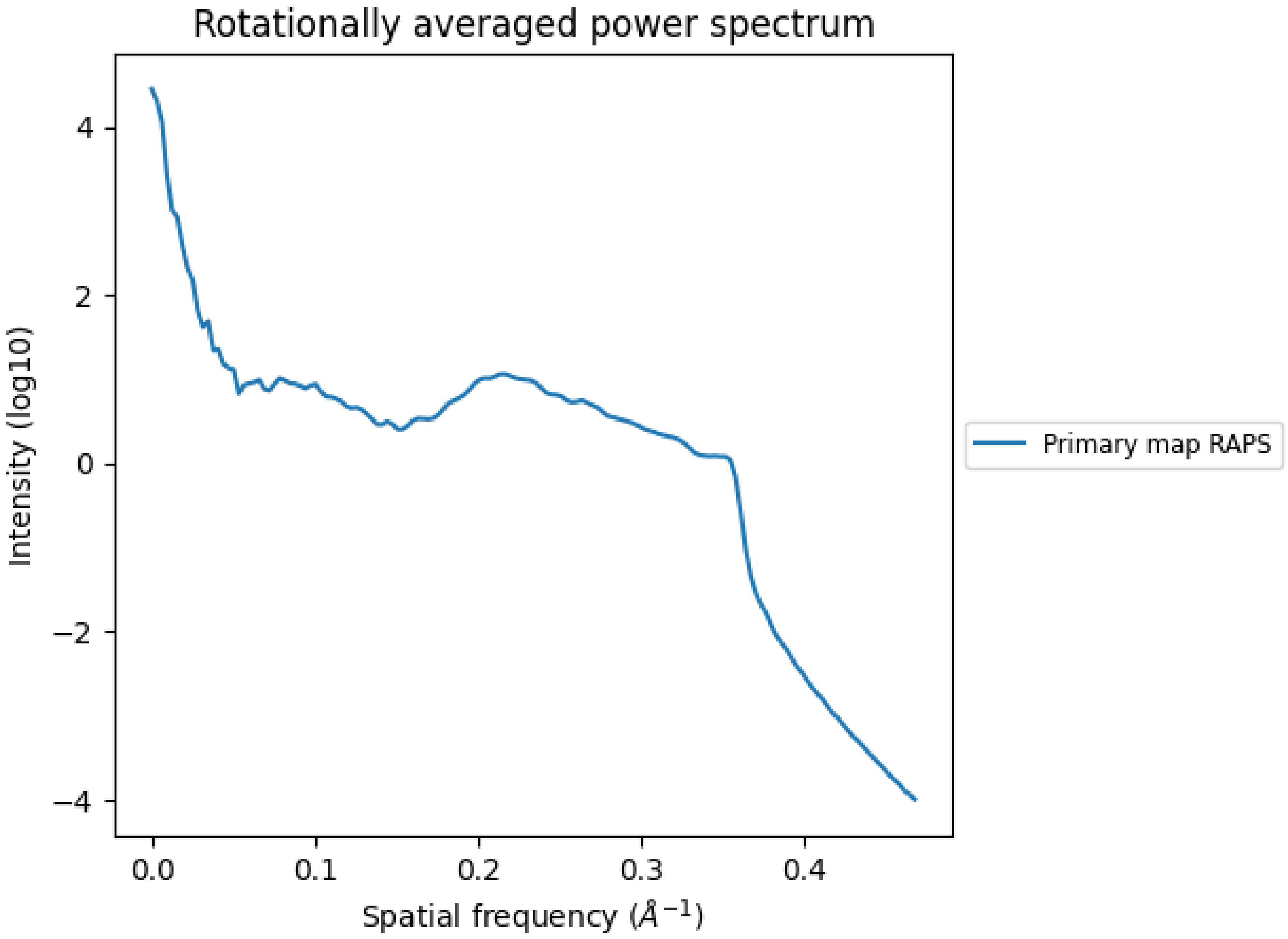

PRELIMINARY

8 Fourier-Shell correlation (i)

Fourier-Shell Correlation (FSC) is the most commonly used method to estimate the resolution of single-particle and subtomogram-averaged maps. The shape of the curve depends on the imposed symmetry, mask and whether or not the two 3D reconstructions used were processed from a common reference. The reported resolution is shown as a black line. A curve is displayed for the half-bit criterion in addition to lines showing the 0.143 gold standard cut-off and 0.5 cut-off.

8.1 FSC (i)

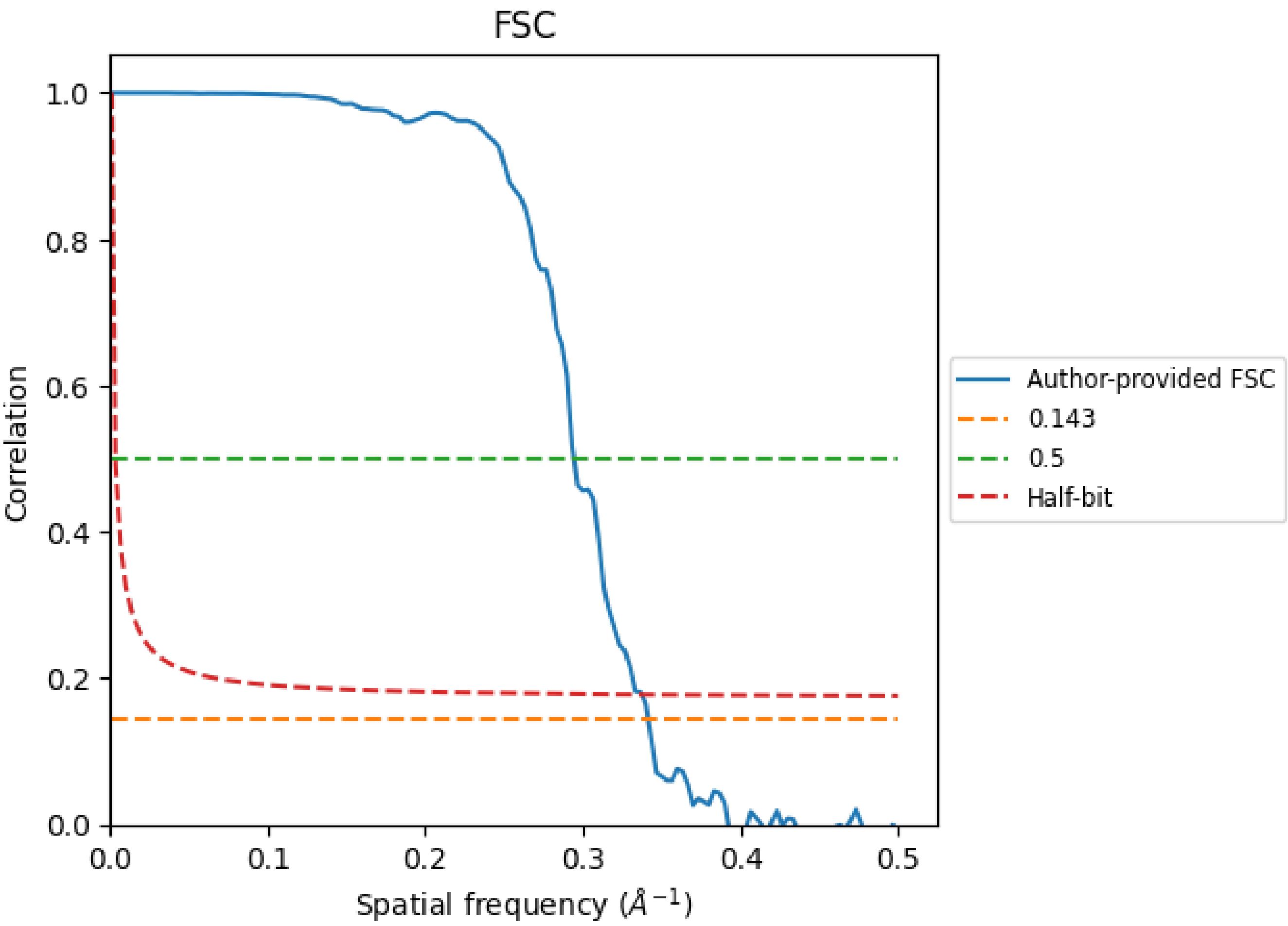

PRELIMINARY REPORT

8.2 Resolution estimates (i)

Resolution estimate (Å)	Estimation criterion (FSC cut-off)		
	0.143	0.5	Half-bit
Reported by author	-	-	-
Author-provided FSC curve	2.93	3.40	2.96
Unmasked-calculated*	-	-	-

*Resolution estimate based on FSC curve calculated by comparison of deposited half-maps.

PRELIMINARY VALIDATION REPORT

9 Map-model fit (i)

This section contains information regarding the fit between EMDB map D_1292118365 and PDB model D_1292118365. Per-residue inclusion information can be found in section 3 on page 5.

9.1 Map-model overlay (i)

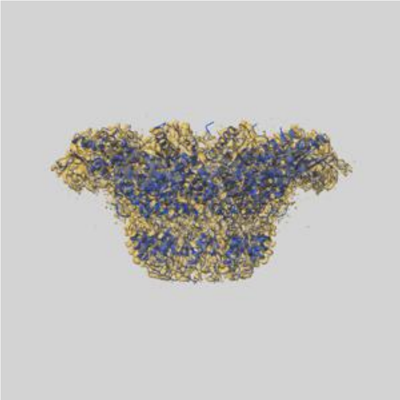

X

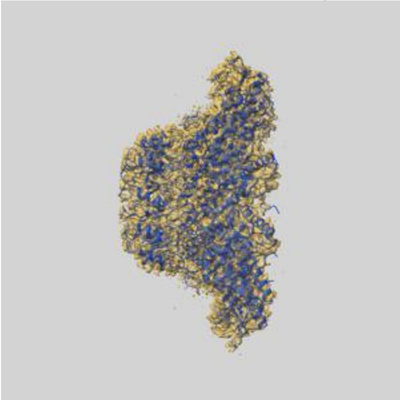

Y

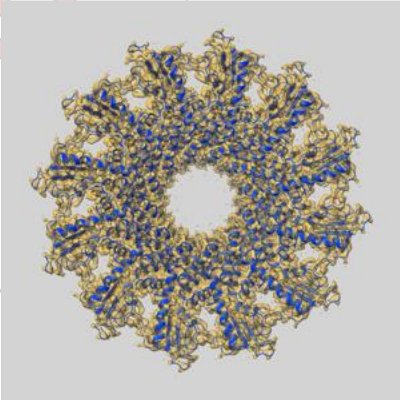

Z

The images above show the 3D surface view of the map at the recommended contour level 0.023 at 50% transparency in yellow overlaid with a ribbon representation of the model coloured in blue. These images allow for the visual assessment of the quality of fit between the atomic model and the map.

PRELIMINARY VALIDATION REPORT

9.2 Atom inclusion (i)

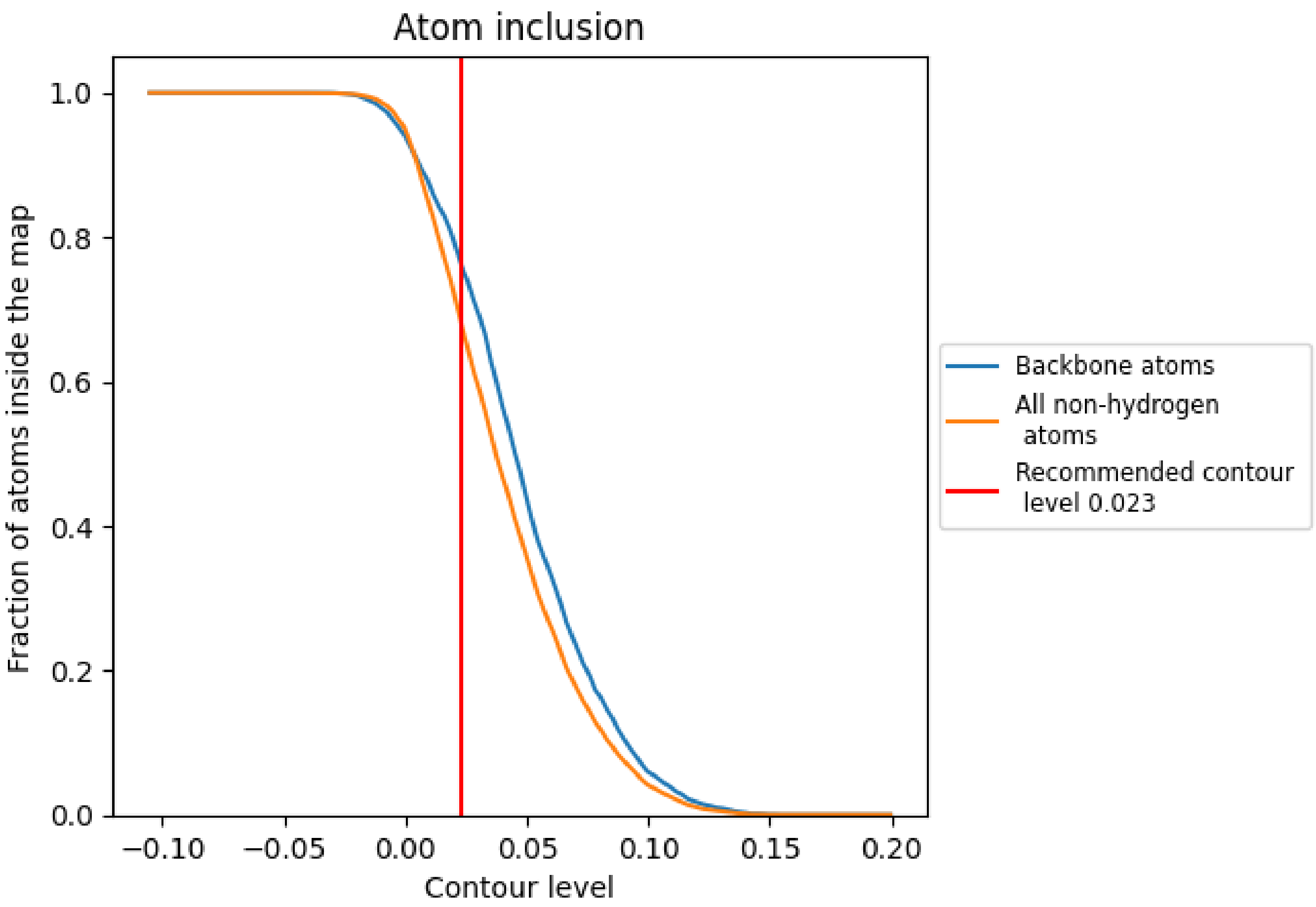

At the recommended contour level, 76% of all backbone atoms, 68% of all non-hydrogen atoms, are inside the map.

PRELIMINARY
