# Supplementary material for: Cryo-EM Structures of Two Bacteriophage Portal Proteins Provide Insights for Antimicrobial Phage Engineering
Source: Viruses. 2021 Dec 16;13(12):2532. doi: 10.3390/v13122532 (PMC8703570; doi:10.3390/v13122532)
Supplement: Supplementary file 1 [file viruses-13-02532-s001.zip › Portals_MS_Supp_fig_015dec21.pdf]

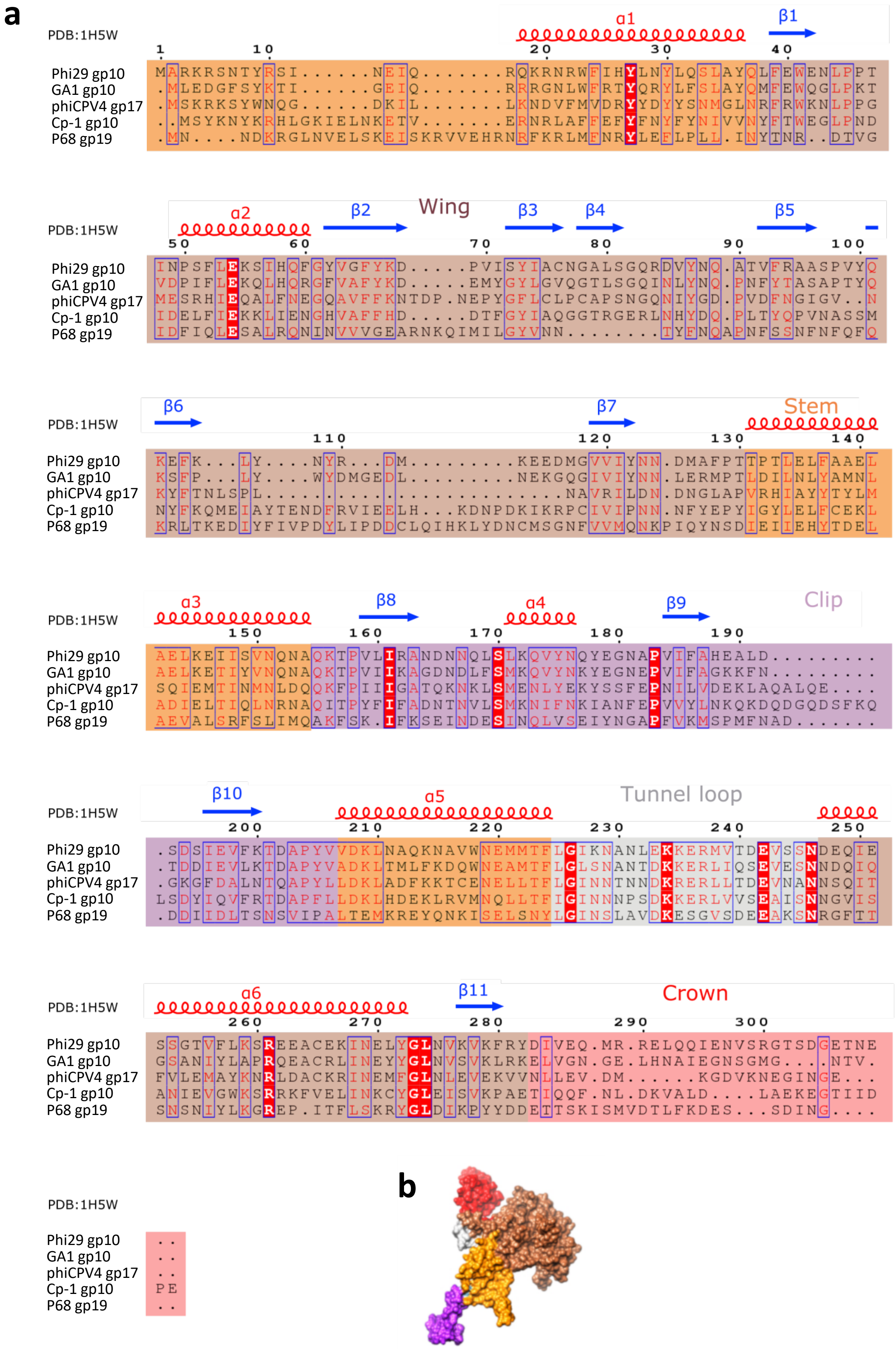

A. Javed et al., Supplementary Figure S1

Supplementary Figure S1. Sequence alignment of 5 portal proteins. a – Residues are indicated using percentage of equivalent similarity as implemented in ESPript 3.0. Conserved residues are highlighted in red. The phi 29 gp10 secondary elements are labelled on top (PDB: 1H5W). The wing, stem and clip domain are designated in brown, orange, and purple colours respectively. The tunnel loop is highlighted in grey and the putative crown domain in pink. **b –** General localisation of domains in space is shown on the SPP1 PP structure (N.B. red space fill on SPP1 PP is equivalent to pink shading at the C-terminus in the MSA).

.

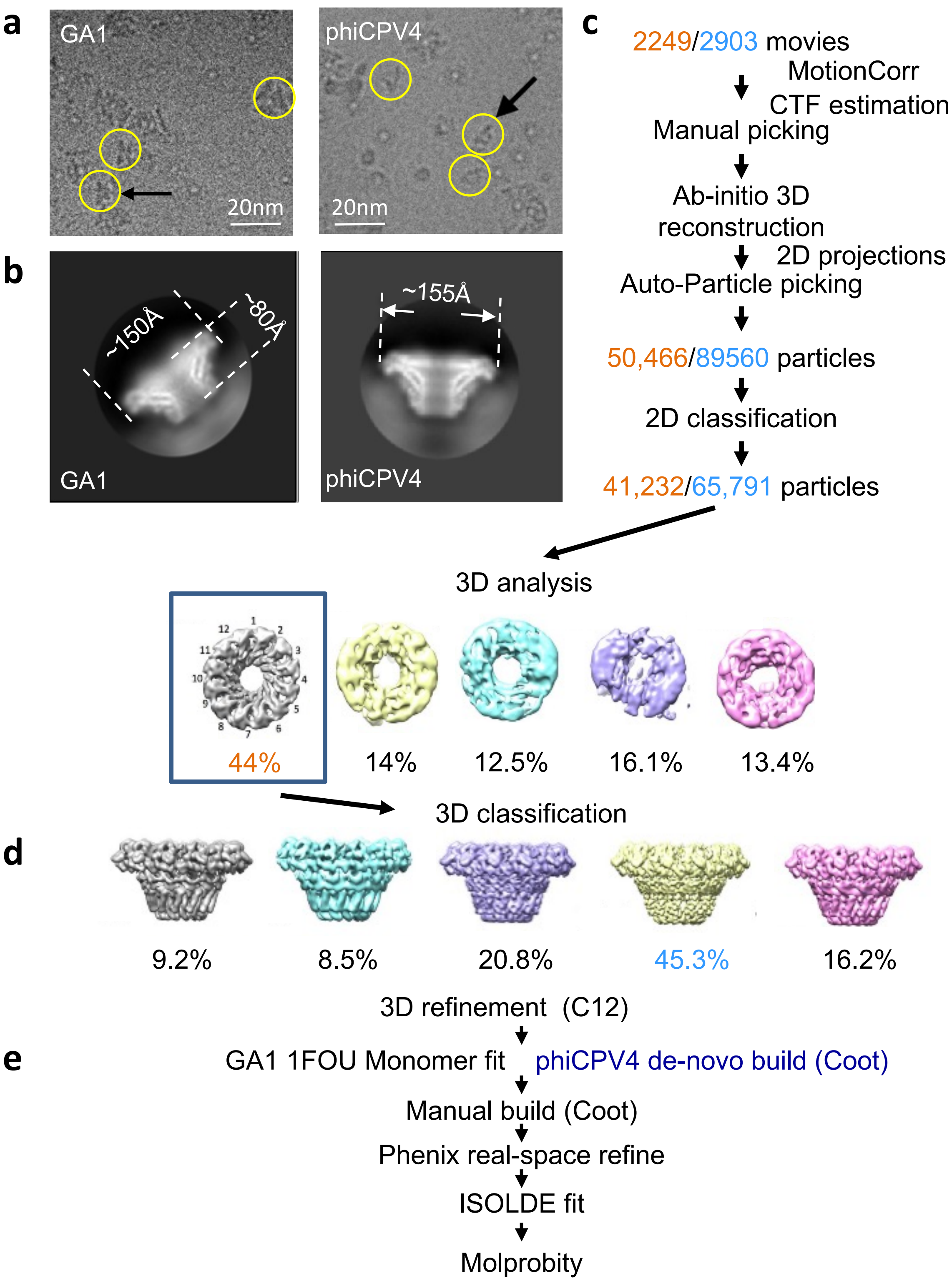

A. Javed et al., Supplementary Figure S2

Supplementary Figure S2. Overall diagram of the image processing protocol. a - Representative cryo-EM micrographs of the GA1 and phiCPV4 PPs. Representative particle images are circled in yellow. **b** – Representative 2D classes (side views) of the portal complexes. **c –** The workflow of the cryo- EM image analysis, arrows link the steps from data collection to 3D reconstructions; the corresponding percentage of particle images comprising each class are indicated. **d –** 3D refinement was done by imposing C12 symmetry followed by 3D classification. **e –** The final steps related to the EM map interpretations by atomic modelling are indicated in the bottom part of the scheme. Validation of the map quality and model fittings was done using a number of different packages (see methods).

a

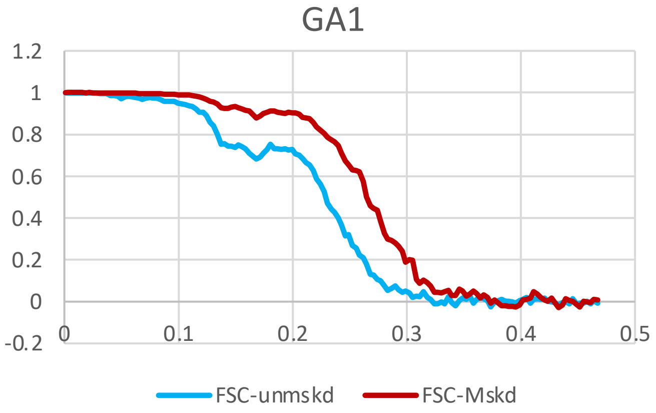

b

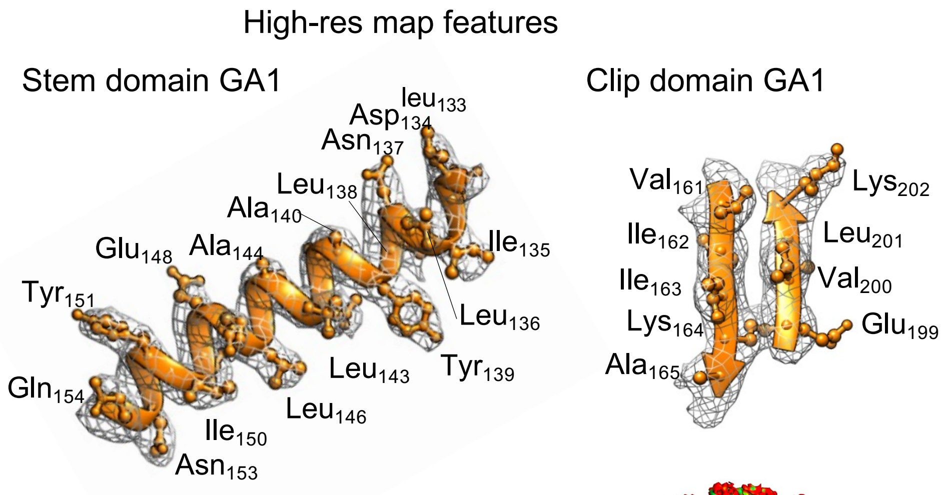

c

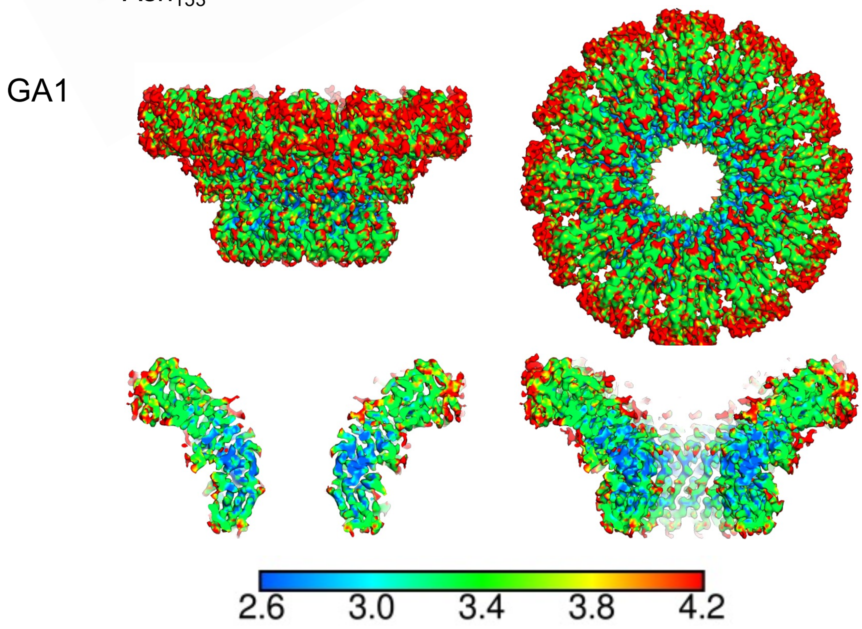

A. Javed et al., Supplementary Figure S3

Supplementary Figure S3. Assessment of GA1 gp10 map resolution. a – Fourier shell correlation (FSC) curves for the GA1 gp10 EM map. The curve in light blue is for the map within a loose mask, the red curve represents the FSC of the map within a tight mask. Representation of the GA1 gp10 EM map coloured according to the local resolution. Regions at higher resolution are in blue, regions at lower in resolution are in red. **b –** Fitting of the atomic models into the EM densities. The left panel shows the fitted α- helix of the stem domain into the EM density (grey mesh). The right panel shows the EM map region corresponding to β-strands of the clip domain. **c –** The map is show from the side, the top, and in the bottom panels are the central slice and cut-away views. The central part of the structure is in blue indicating higher resolution. The periphery (edges of wings) is seen in red, where the resolution is lower due to the higher flexibility of peripheral structural elements.

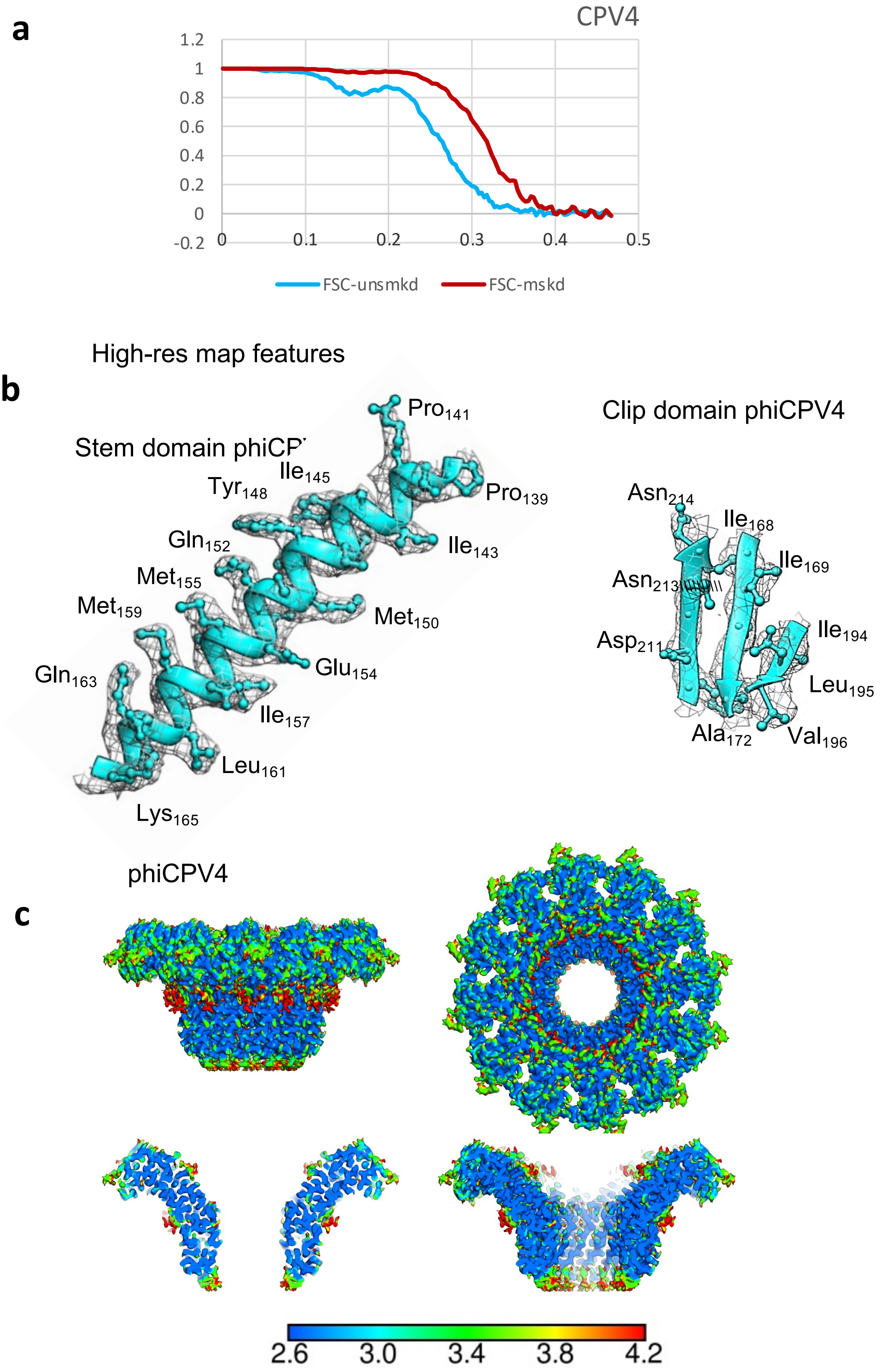

A. Javed et al., Supplementary Figure S4

Supplementary Figure S4. Assessment of phiCPV4 gp17 map resolution. a – Fourier shell correlation (FSC) curves for the phiCPV4 gp17 EM map. The curve in light blue is for the map within a loose mask, the red curve represents the FSC of the map within a tight mask. **b –** Fitting of the atomic models into the EM densities. The left panel shows the fitted α-helix of the stem domain into the EM density (grey mesh). The right panel shows the EM map region corresponding to β-strands of the clip domain. **c –** Representation of the phi CPV4 gp17 EM map coloured according to the local resolution. Regions at higher resolution are in blue, regions at lower in resolution are in red. The map is shown from the side, the top, and in the bottom panels are the central slice and cut-away views. The central part of the structure is in blue indicating higher resolution. The periphery (the area of the N-terminus) is seen in red, where the resolution is lower due to the higher flexibility of peripheral structural elements.

Clip domains

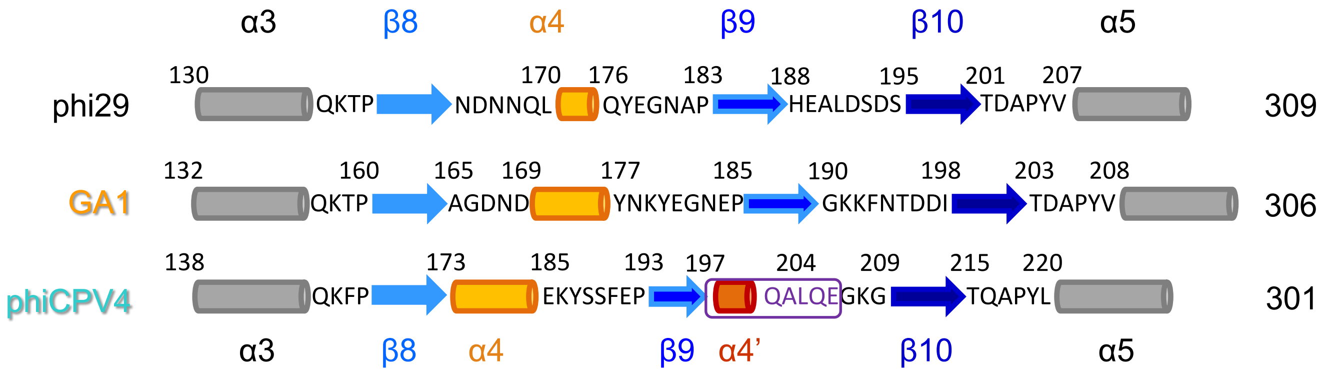

Supplementary Figure S5. Secondary structure alignment of the clip domain of the polypeptide chain, α3–α5. Alignment for phi29, GA1 and phiCPV4 is based on the results of fitting of the atomic models. The length of the cylinders (α-helices) and arrows (β-strands) are approximately proportional to the sizes of these elements in the structures. In purple is shown the extra insertion within the clip domain of phiCPV4 gp17.

.
