# Supplementary material for: Cryo-EM Structures of Two Bacteriophage Portal Proteins Provide Insights for Antimicrobial Phage Engineering
Source: Viruses. 2021 Dec 16;13(12):2532. doi: 10.3390/v13122532 (PMC8703570; doi:10.3390/v13122532)
Supplement: Supplementary file 1 [file viruses-13-02532-s001.zip › Supplementary_Table_1_EM-data_07dec21.pdf]

Supplementary Table S1. Summary of structural evaluation of Portal complexes.

	GA1	PhiCPV4
**EMDB, PDB**	EMD-13664, 7PV2	EMD-13665, 7PV4
Microscope (Voltage)	Titan Krios (300kV)	Titan Krios (300kV)
Pixel size (Å/pix.)	1.07	1.07
Camera	K2 (Counting)	K2 (Counting)
Defocus range (μm)	-1.2 – 2.0	-1.2 – 2.0
Electron Dose (e-/ $Å^2$/frame)	0.98	0.985
Symmetry	C12	C12
Number of micrographs	2249	2903
Initial number of particles	50,466	89,560
Final number of particles	22,456	26,940
Map resolution range Å	3.0 – 6.0	2.8 – 6.0
Map Sharpening B Factor	-97.7	-74.6
**Refinement**		
Initial Model (PDB)	1FOU	I-Tasser
Model Resolution Å	3.2	2.8
**Model composition (Monomer)**		
Non-hydrogen atoms	0	0
Protein residues	257	256
Nucleotide	0	0
**R.m.s Deviations**		
Bond Lengths	0.011	0.012
Bond angles	0.714	2.05
**Validation**		
Molprobity Score	1.48	0.79
Clashscore	4.28	0.5
**Ramachandran (%)**		
Favoured (%)	96.1	98.4
Allowed (%)	3.95	1.19
Outliers (%)	0	0.4
